# Supplementary material for: CRISPRbuilder-TB: “CRISPR-builder for tuberculosis”. Exhaustive reconstruction of the CRISPR locus in mycobacterium tuberculosis complex using SRA
Source: PLoS Comput Biol. 2021 Mar 5;17(3):e1008500. doi: 10.1371/journal.pcbi.1008500 (PMC7968741; doi:10.1371/journal.pcbi.1008500)
Supplement: S3 Table — CRISPR features derived from complete genome sequences using Spolpred-like tool. (DOCX) [file pcbi.1008500.s003.docx]

**S3 Table. CRISPR-Cas features of reference strains according to complete genome sequences and according to CRISPR-builder run on WGS-data.**  CRISPR features derived from complete genome sequences using Spolpred-like tool

| Name | **Accession.version** (GenBank Assembly ID) | Lineage  (SNP-based) | **Group** (spoligo-based) | SIT | Spoligotype pattern ("ancient", 43-spacers based) | Submitter | Date |
| --- | --- | --- | --- | --- | --- | --- | --- |
| H37Ra | GCA_001938725.1 | 4; 4.9 | Euro-American-PGG3 | 451 | ■■■■■■■■■■■■■■■■■■■□□■■■■■■■■■■■□□□□■■■■■■■ | San Diego State Univ. | 06/01/2017 |
| CDC1551 | GCA_000008585.1 | 4; 4.1; 4.1.1; 4.1.1.3 | Euro-American-PGG2 | 549 | ■■■□□□□□□□□□■■■■■□■■■■□■■■■■■■■■□□□□■■■■■■■ | TIGR | 04/08/2004 |
| Erdman = ATCC 35801 | GCA_000350205.1 | 4; 4.1; 4.1.2; 4.1.2.1 | Euro-American-PGG2-Haarlem | 47 | ■■■■■■■■■■■■■■■■■■■■■■■■■□□□□□□■□□□□■■■■■■■ | Nat. Center Global Health and Medicine | 01/03/2012 |
| F11 | GCA_000016925.1 | 4; 4.3; 4.3.2; 4.3.2.1 | Euro-American-PGG2-LAM | ND | ■■■■■■■■□□□■■■■■■■■■■□□□■■■■■■■■□□□□■■■■■■■ | Broad Institute | 07/06/2007 |
| W-148 | GCA_000193185.2 | 2; 2.2; 2.2.1; 2.2.1.2; 2.2.1.2.2; 2.2.1.2.2.3.1 | Beijing | 1 | □□□□□□□□□□□□□□□□□□□□□□□□□□□□□□□□□□■■■■■■■■■ | Broad Institute | 19/08/2015 |
| BCG str. Pasteur 1173P2 | GCA_000009445.1 | BOV; BOV_AFRI | M. bovis | 482 | ■■□■■■■■□■■■■■■□■■■■■■■■■■■■■■■■■■■■■■□□□□□ | M. bovis sequencing teams | 08/01/2007 |
| BCG str. Tokyo 172 | GCA_000010685.1 | BOV; BOV_AFRI | M. bovis | 482 | ■■□■■■■■□■■■■■■□■■■■■■■■■■■■■■■■■■■■■■□□□□□ | Japan BCG Laboratory | 09/03/2009 |

CRISPR-Cas features derived from WGS runs using CRISPRbuilder-TB

| Name | Accession | **Lineage** (SNP-based) | **Group** (spoligo-based) | SIT | Cas-genes  set | Spoligotype pattern ("new" 68-spacers based) |  |
| --- | --- | --- | --- | --- | --- | --- | --- |
| H37Ra | SRR6407486 | 4; 4.9 | Euro-Am. GG3 | 451 | ■■■■■■■■■ | ■■■■□□□□□□□■■■■□□■■■■■■■■■■■■□□■■■■■■■■■■□■□□□□□□□□■■■□□□□□□□□■■■■■■■ |  |
| CDC1551 | SRR1051196 | 4; 4.1; 4.1.1; 4.1.1.3 | Euro-Am PGG2 | 549 | ■■■■■■■■■ | ■■■■□□□□□□□□□□□□□□□□□□■■■■■□■■■■□■■■■■■■■■■□□□□□□□□■■■□□□□□□□□■■■■■■■ |  |
| Erdman = ATCC 35801 | SRR1011525 | 4; 4.1; 4.1.2; 4.1.2.1 | Euro-Am PGG2-Haarlem | 47 | ■■■■■■*^839^*IS■■■ | ■■■■□□□□□□□■■■■□□■■■■■■■■■■■■■■■■■□□□□□□□■■□□□□□□□□■■■□□□□□□□□■■■■■■■ |  |
| F11 | SRR974839 | 4; 4.3; 4.3.2; 4.3.2.1 | Euro-Am PGG2-LAM | ND | ■■■■■■■■■ | ■■■■□□□□□□□■■■■□□■□□□■■■■■■■■■■^25^□□□■■■■■■■□■□□□□□□□□■■■□□□□□□□□■■■■■■■ |  |
| W-148 | SRR849475 | 2; 2.2; 2.2.1; 2.2.1.2; 2.2.1.2.2; 2.2.1.2.2.3.1 | Beijing | 1 | ■■■■■^341^□□□□ | □□□□□□□□□□□□□□□□□□□□□□□□□□□□□□□□□□□□□□□□□□□□□□■■■■■■■■□□□□□□□□■■■■■■■ |  |
| BCG str. Pasteur 1173P2 | SRR1915486 | BOV ; BOV_AFRI | M. bovis | 482 | ■■■■■■■■■ | ■■■□■■■■□□■■■■■■■■□■■■■■■□■■■■■■■■■■■■■■■■■■■□■■■□■■■□□□□□□□□□□□□□□□□ |  |
| BCG str. Tokyo 172 | DRR029469 | BOV; BOV_AFRI | M. bovis | 482 | ■■■■■■■■■ | ■■■□■■■■□□■■■■■■■■□■■■■■■□■■■■■■■■■■■■■■■■■■■□■■■□■■■□□□□□□□□□□□□□□□□ |  |

Foot-note of Table 2: The filled squares correspond to presence, empty squares to absence of sequences of interest. The black squares correspond to Direct Variant Repeats (DVR *i.e.* DR+spacer) with the known spacers used in the 43-spacers standard spoligotype format, in their classical order (which matches genome order). The red square corresponds to a DVR with a mutation of 4 SNPs as compared to H37Rv sequence. Truncated DR around spacers 24 and 25 (old-format *i.e.* spacers 34 and 35 in the “new” 68 spacers format) around IS*6110* insertion were not distinguished from standard ones. The blue squares correspond to DVR with spacers included in the 68- and not in the 43-spacers spoligotype-format. The underlined DVR is a repetition of DVR35 after DVR41. The orange squares correspond to Cas genes in their genome order. Number in exponent indicate the number of nucleotides of the gene or spacer that is present before a deletion or an IS*6110*. insertion. Note that, apart from the fact that CRISPR-builder provides more information than Spolpred tools applied on complete genome sequences, all information is concordant between the two tables except for H37Ra where complete genome describes a variant for spacer 13 (probably as an artifact, see text).
