# Supplementary material for: CRISPRbuilder-TB: “CRISPR-builder for tuberculosis”. Exhaustive reconstruction of the CRISPR locus in mycobacterium tuberculosis complex using SRA
Source: PLoS Comput Biol. 2021 Mar 5;17(3):e1008500. doi: 10.1371/journal.pcbi.1008500 (PMC7968741; doi:10.1371/journal.pcbi.1008500)
Supplement: S6 Table — (DOCX) [file pcbi.1008500.s006.docx]

**S6 Table. CRISPR-Cas locus profile reconstructed from public WGS runs and representative of MTC diversity**

| Accession | Lineage according to SNPs | Cas6 | Csm1 | Csm2 | Csm3 | Csm4 | Csm5 | Csm6 | Cas1 | Cas2 | Full-length spoligotypes |
| --- | --- | --- | --- | --- | --- | --- | --- | --- | --- | --- | --- |
| ERR234156 | 1; 1.1;  1.1.1 | ■ | ■ | ■ | ■ | ■ | ■ | ■ | ■ | ■ | ■■■■■■■■■■■■■■■■■■■■■■■■■■■■■■■■■■■■■■□□□□■□■■■□■■■■■■■■■■■■■■■■■■■■□□□□□□□□□□□□□□□□□□□□□□□□□□□□□□ |
| ERR036222 | 1; 1.1; 1.1.3 | ■ | ■ | ■ | ■ | ■ | ■ | ■ | ■ | ■ | ■■■■■■□□□□■□□□□□□□□■■■■■■■■■■■■■□□■■■■□□□□■□■■■□■■■■■■■□■■■■■■■■■■■■□□□□□□□□□□□□□□□□□□□□□□□□□□□□□□ |
| ERR751771 | 1; 1.2.1; 1.2.1.1 | ■ | ■ | ■ | ■ | ■ | ■ | ■ | ■ | ■ | ■■■□■■■■■□■■■■■■■■■■■■■■■■■■■□□■■■■■■■□□□□■□■■■□■■■■■■■■■■■■■■■■■■■■□□□□□□□□□□□□□□□□□□□□□□□□□□□□□□ |
| ERR234164 | 1; 1.2.2 | ■ | ■ | ■ | ■ | ■ | ■ | ■ | ■ | ■ | ■■■■■■■■■■■■■■■■■■■■■■■■■■■■■■■■■■■■■■□□□□■□■■■□■■■■■■■■■■■■■□■■■■■■□□□□□□□□□□□□□□□□□□□□□□□□□□□□□□ |
| SRR1710060 | 2; 2.1 | ■ | ■ | ■ | ■ | ■ | ■ | _1021_ ■ | ■ | ■ | ■■■■■■■■■■■■■■■□□■■■■■■■■■■■■■■■■■■■■■■■■■■■■■■■■■■■■□□□□□□□□■■■■■■■□□□□□□□□□□□□□□□□□□□□□□□□□□□□□□ |
| ERR234252 | 2; 2.1 | ■ | ■ | ■ | ■ | ■ | ■ | _1021_ ■ | □ | □ | □□□□□□□□□□□□□□□□□□□□□□□□□□□□□□□□□□■■■■■■■■■■■■■■■■■■■□□□□□□□□■■■■■■■□□□□□□□□□□□□□□□□□□□□□□□□□□□□□□ |
| ERR551636 | 2; 2.2; 2.2.2 | ■ | ■ | ■ | ■ | _341_ ■ | □ | □ | □ | □ | □□□□□□□□□□□□□□□□□□□□□□□□□□□□□□□□□□□□□□□□□□□□□■■■■■■■■□□□□□□□□■■■■■■■□□□□□□□□□□□□□□□□□□□□□□□□□□□□□□ |
| ERR234109 | 3 | ■ | ■ | ■ | ■ | ■ | ■ | ■ | ■ | ■ | ■■■■■■■■■■□□□□□□□■■■■■■■■■■■■■■■□□□□□□□□□□□□■■■■■■■■■□□□□□□□□■■■■■■■□□□□□□□□□□□□□□□□□□□□□□□□□□□□□□ |
| ERR2245388 | 3; 3.1.1 | ■ | ■ | ■ | ■ | ■ | ■ | ■ | ■ | ■ | ■■■■■■■■■■□□□□□□□■■□■■■■■■■■■□□□□□□□□□□□□□□□□□■■■■■■■□□□□□□□□■■■■■■■□□□□□□□□□□□□□□□□□□□□□□□□□□□□□□ |
| ERR234192 | 3; 3.1.2; 3.1.2.1 | ■ | ■ | ■ | ■ | ■ | ■ | ■ | ■ | ■ | ■■■■■■■■■□□□□□□□□□□□■■■■■■■■■■■■■□□□□□□□□□□□■■■■■■■■■□□□□□□□□■■■■■■■□□□□□□□□□□□□□□□□□□□□□□□□□□□□□□ |
| ERR2652972 | 4; 4.1; 4.1.2 | ■ | ■ | ■ | ■ | ■ | ■ | ■ | ■ | ■ | ■■■■□□□□□□□■■■■□□■■■■■□■■■■■■■■■■■■■■■■■■■□□□□□□□□■■■□□□□□□□□■■■■■■■□□□□□□□□□□□□□□□□□□□□□□□□□□□□□□ |
| ERR067645 | 4; 4.2; 4.2.1 | ■ | ■ | ■ | ■ | ■ | ■ | ■ | ■ | ■ | ■■■■□□□□□□□■■■■□□□□■■■■■■■■■■■■■■■■■■■□□□■□□□□□□□□■■■□□□□□□□□■■■■■■■□□□□□□□□□□□□□□□□□□□□□□□□□□□□□□ |
| ERR234258 | 4; 4.3; 4.3.3 | ■ | ■ | ■ | ■ | ■ | ■ | ■ | ■ | ■ | ■■■■□□□□□□□■■■■□□■■■■■■■■■□□□□□■■■■□□□□■■■□□□□□□□□■■■□□□□□□□□■■■■■■■□□□□□□□□□□□□□□□□□□□□□□□□□□□□□□ |
| SRR5073887 | 4; 4.4; 4.4.1; 4.4.1.1 | ■ | ■ | ■ | ■ | ■ | ■ | _439_ ■ | ■ | ■ | ■■■■□□□□□□□■■■■□□■□□■■■■■■■■■■■■■■■■■■■■■■□□□□□□□□■■■□□□□□□□□■■■■□□■□□□□□□□□□□□□□□□□□□□□□□□□□□□□□□ |
| SRR5073715 | 4; 4.5 | ■ | ■ | ■ | ■ | ■ | ■ | ■ | ■ | ■ | ■■■■□□□□□□□■■■■□□■■■■■■■■■■■■■■■■■■■■■■■■■□□□□□□□□■■■□□□□□□□□■■■■■■■□□□□□□□□□□□□□□□□□□□□□□□□□□□□□□ |
| ERR551416 | 4; 4.6; 4.6.1; 4.6.1.1 | ■ | ■ | ■ | ■ | ■ | ■ | ■ | ■ | ■ | ■■■□□□□□□□□□■■■□□■■■■■■■■■□□■■■■■■■■■■■■■■□□□□□□□□■■■□□□□□□□□□■■□□■■□□□□□□□□□□□□□□□□□□□□□□□□□□□□□□ |
| ERR2652992 | 4; 4.7 | ■ | ■ | ■ | ■ | ■ | ■ | ■ | ■ | ■ | ■■■■□□□□□□□■■■■□□■■■■■■■■■■■□□□□□□□□■■■■■■□□□□□□□□■■■□□□□□□□□■■■■■■■□□□□□□□□□□□□□□□□□□□□□□□□□□□□□□ |
| ERR2652941 | 4; 4.9 | ■ | ■ | ■ | ■ | ■ | ■ | ■ | ■ | ■ | ■■■■□□□□□□□□□■■□□■■■■■■■■■■■■■■■■■■■■■■■■■□□□□□□□□■■■□□□□□□□□■■■■■■■□□□□□□□□□□□□□□□□□□□□□□□□□□□□□□ |
| ERR1971863 | 7 | ■ | ■ | ■ | ■ | ■ | ■ | ■ | ■ | ■ | ■■■■■■□□■■■□□□□□□□□□□□□□□□□□□□□□□□■■■□□■■■■■■■■■■□■■■□□□□□□□□■■■■■■■□□□□□□□□□□□□□□□□□□□□□□□□□□□□□□ |
| ERR751300 | 5 | ■ | ■ | ■ | ■ | ■ | ■ | ■ | ■ | ■ | ■■■■■■■■■■■■■■■■■□□□□□■■■■■■■■■■■■■■■■■■■■■■■■■■■■□□□□□□□□□□■■■■■■■■□□□□□□□□□□□□□□□□□□□□□□□□□□□□□□ |
| SRR998631 | 6; BOV_AFRI | ■ | ■ | ■ | ■ | ■ | ■ | ■ | ■ | ■ | ■■■■■■■■■■■■■■□□□□□■■■■■■■■■■■■■■■■■■■■■■■□■■■■■■■■■□□□□□■■■■■■■■■■■□□□□□□□□□□□□□□□□□□□□□□□□□□□□□□ |
| ERR502499 | M. bovis | ■ | ■ | ■ | ■ | ■ | ■ | ■ | ■ | ■ | ■■■□■■■■□□■■■■■■■■□■■■■■■□■■■■■■■■■■■■■□□□■■■■■■□■■■□□□□□□□□□□□□□□□□□□□□□□□□□□□□□□□□□□□□□□□□□□□□□□ |
| ERR1462634 | M. caprae | ■ | ■ | ■ | ■ | ■ | ■ | ■ | ■ | ■ | □□■□□□□□□□□□□□□□□□□□□□□□□□■■■■■■■■■■■□■■■■■■■■■■■■■■□□□□□□□□□□□□□□□□□□□□□□□□□□□□□□□□□□□□□□□□□□□□□□ |
| ERR1336822 | M. canettii |  |  |  |  |  |  |  | ■ |  | □□□□□□□□□□□□□□□□□□□□□□□□□□□□□□□□□□□□□□□□□□□□□□□□□□□□□□□□□□□□□□□□□□□□■■■■■■■■■■■■■■■■■■■■■■■■■■■■■■ |
