# Supplementary material for: CRISPRbuilder-TB: “CRISPR-builder for tuberculosis”. Exhaustive reconstruction of the CRISPR locus in mycobacterium tuberculosis complex using SRA
Source: PLoS Comput Biol. 2021 Mar 5;17(3):e1008500. doi: 10.1371/journal.pcbi.1008500 (PMC7968741; doi:10.1371/journal.pcbi.1008500)
Supplement: S8 Table — (DOCX) [file pcbi.1008500.s008.docx]

**S8 Table. DR and spacer variants for the representative set of MTC diversity**

| Accession | Lineage according to SNPs |  | 4*DR7*5 | 8*DR11*9 | 14*DR1*15 | 29*DR21*32 | 30*DR2*31 | 44*DR26*45 | 46*DR15*47 | 49*DR27*50 | 50*DR3*51 | 64*DR6*65 | 66*DR4*67 | 67*DR5*68 |  | sp4_var | sp6_var | sp38_var | sp60_var | sp82_var |
| --- | --- | --- | --- | --- | --- | --- | --- | --- | --- | --- | --- | --- | --- | --- | --- | --- | --- | --- | --- | --- |
| ERR234156 | 1; 1.1; 1.1.1 |  | □ | □ | ■ | . | ■ | . | □ | □ | ■ | □ | ■ | ■ |  |  |  | 1 |  |  |
| ERR036222 | 1; 1.1; 1.1.3 |  | □ | . | . | . | ■ | . | □ | □ | ■ | □ | ■ | ■ |  |  |  |  |  |  |
| ERR751771 | 1; 1.2.1; 1.2.1.1 |  | . | □ | □ | ■ | . | . | □ | □ | ■ | □ | ■ | ■ |  |  |  |  | 1 |  |
| ERR234164 | 1; 1.2.2 |  | □ | □ | □ | . | ■ | . | □ | □ | ■ | □ | ■ | ■ |  |  |  |  |  |  |
| SRR1710060 | 2; 2.1 |  | □ | □ | □ | . | ■ | □ | □ | □ | □ | ■ | ■ | ■ |  |  |  |  |  |  |
| ERR234252 | 2; 2.1 |  | . | . | . | . | . | □ | □ | □ | □ | ■ | ■ | ■ |  |  |  |  |  |  |
| ERR551636 | 2; 2.2; 2.2.2 |  | . | . | . | . | . | . | □ | □ | □ | ■ | ■ | ■ |  |  |  |  |  |  |
| ERR234109 | 3 |  | □ | ■ | . | . | ■ | . | □ | □ | □ | ■ | ■ | ■ |  |  |  |  |  |  |
| ERR2245388 | 3; 3.1.1 |  | □ | □ | . | . | . | . | . | □ | □ | ■ | ■ | ■ |  |  |  |  |  |  |
| ERR234192 | 3; 3.1.2; 3.1.2.1 |  | □ | □ | . | . | ■ | . | □ | □ | □ | ■ | ■ | ■ |  |  |  |  |  |  |
| ERR2652972 | 4; 4.1; 4.1.2 |  | . | . | □ | . | ■ | . | . | . | . | ■ | ■ | ■ |  |  |  |  |  |  |
| ERR067645 | 4; 4.2; 4.2.1 |  | . | . | □ | . | ■ | . | . | . | . | ■ | ■ | ■ |  |  |  |  |  |  |
| ERR234258 | 4; 4.3; 4.3.3 |  | . | . | □ | . | . | . | . | . | . | ■ | ■ | ■ |  |  |  |  |  |  |
| SRR5073887 | 4; 4.4; 4.4.1; 4.4.1.1 |  | . | . | □ | . | ■ | . | . | . | . | ■ | . | . |  |  |  |  |  |  |
| SRR5073715 | 4; 4.5 |  | . | . | □ | . | ■ | . | . | . | . | ■ | ■ | ■ |  |  |  |  |  |  |
| ERR551416 | 4; 4.6; 4.6.1; 4.6.1.1 |  | . | . | □ | . | ■ | . | . | . | . | ■ | . | ■ |  |  |  |  |  |  |
| ERR2652992 | 4; 4.7 |  | . | . | □ | . | . | . | . | . | . | ■ | ■ | ■ |  |  |  |  |  |  |
| ERR2652941 | 4; 4.9 |  | . | . | . | . | ■ | . | . | . | . | ■ | ■ | ■ |  |  |  |  |  |  |
| ERR1971863 | 7 |  | ■ | . | . | . | . | □ | □ | . | . | □ | ■ | ■ |  |  | 1 |  |  |  |
| ERR751300 | 5 |  | □ | □ | □ | . | ■ | □ | □ | □ | . | □ | ■ | ■ |  |  |  |  |  |  |
| SRR998631 | 6; BOV_AFRI |  | □ | □ | . | . | ■ | □ | □ | □ | □ | □ | ■ | # |  | 2 |  |  |  |  |
| ERR502499 | M. bovis |  | . | . | □ | . | ■ | □ | ■ | . | □ | . | . | . |  |  |  |  |  |  |
| ERR1462634 | M. caprae |  | . | . | . | . | ■ | ■ | ■ | ■ | □ | . | . | . |  |  |  |  |  |  |
| ERR1336822 | M. canettii |  | . | . | . | . | . | . | . | . | . | . | . | . |  |  |  |  |  | 1 |
