# Supplementary material for: CRISPRbuilder-TB: “CRISPR-builder for tuberculosis”. Exhaustive reconstruction of the CRISPR locus in mycobacterium tuberculosis complex using SRA
Source: PLoS Comput Biol. 2021 Mar 5;17(3):e1008500. doi: 10.1371/journal.pcbi.1008500 (PMC7968741; doi:10.1371/journal.pcbi.1008500)
Supplement: S1 Text — (PDF) [file pcbi.1008500.s009.pdf]

Simulation of additional CRISPRs

For each CRISPR (4 to 6), the sequence and characteristics of the CRISPR are provided (I), the reconstruction by the different tools is then presented (CRASS in II, CRISPR_detector in III, CRISPRbuilder-TB in IV). Corresponding schematic views are provided in S4.

A -Simulation and reconstruction of CRISPR, CRISPR-4

A.I. Simulated data

Evolution characteristics :
0.5% mutation.
Tandem duplications allowed.
IS6110 insertions allowed.

Length reads : 75.

Nucleotidic sequence after evolution:
CATCATCAGCAGGCATTGTTACCACACGCTGGACGAATTGTCCATAGAGTCGTCAGACCCAAAACCCCGAGAGGGGAC
GGAAACTTAAAACCGTGTTGCACTGCAACCCGGAATTCTTGCACGTCGTCAGACCCAAAACCCCGAGAGGGGACGGA
AACCATAGAGGGTCGCCGGCTCTGGATCACGCTCCCCTAGTCGTGTCGTCAGACCCAAAACCCCGAGAGGGGACGGA
AACTTTTTGCCTCATGCTTGGGCGACAGCTTTTGACCAAGTCGTCAGACCCAAAACCCCGAGAGGGGACGGAAACTC
GCAAGCGCCGTGCTTCCAGTGATCGCCTTCTAGTCGTCAGACCCAAAACCCCGAGAGGGGACGGAAACTCGCGGCG
CGGCATGGCACGGCAGGCGTGGCTAGGGGGTCGTCAGACCCAAAACCCCGAGAGGGGACGGAAACATGTGCGCCG
TCGCCGTAAGTGCCCCACGGCCCGTGTCGTCAGACCCAAAACCCCGAGAGGGGACGGAAACATTTCGACGACAATTC
GTTGACCACGAATTTTCAGAGTCGTCAGACCCAAAACCCCGAGAGGGGACGGAAACACATCCCACGCGTTACCGCTG
GCGCGCATCATTCATCGAGTCGTCAGACCCAAAACCCCGAGAGGGGACGGAAACCCATATCGGGGACGGCGACGCT
GCGAGAGGACACGCCGAGTCGTCAGACCCAAAACCCCGAGAGGGGACGGAAACTACACCACGCGTCGTGCCATCAG
TCAGCGTCCTCCTCGTCGTCAGACCCAAAACCCCGAGAGGGGACGGAAACTTGAACACGGAGCCTTGCACATGCCGT
GGCTCAGGGGTGTCGTCAGACCCAAAACCCCGAGAGGGGACGGAAACAACACCTCAGTAGCACGTCATACGCCGAC
CAATCATCAGGTCGTCAGACCCAAAACCCCGAGAGGGGACGGAAACTTTTCTGACCACTTGTGCGGGATTAGCGGGC
TTAGGTCGTCAGACCCAAAACCCCGAGAGGGGACGGAAACACCAATGCGTCGTCATTTCCGGCTTCAATTTCAGCCTG
TCGTCAGACCCAAAACCCCGAGAGGGGACGGAAACCTGAGGAGAGCGAGTACTCGGGGCTGCCGTCTGCGCTGGTC
GTCAGACCCAAAACCCCGAGAGGGGACGGAAACACGACGTTAGGGCATGCAGCATGCCGTCCCCGTTTTTGAGTCGT
CAGACCCAAAACCCCGAGAGGGGACGGAAACTGCTCTTGAGCAACGCCATCATCCGGCGCCGCAGCTCCGCGTCGT
CAGACCCAAAACCCCGAGAGGGGACGGAAACGCGTGAAACCGCCCCCAGCCTCGCCGGGGCCGCCTAGGTCGTCA
GACCCAAAACCCCGAGAGGGGACGGAAACACTCGGAATCCCATGTGCTGACAGCGGATTCGCATGTCGTCAGACCCA
AAACCCCGAGAGGGGACGGAAACCGGGCAGCGTTCGACACCCGCTCTAGTTGACTTCCGGGTCGTCAGACCCAAAA
CCCCGAGAGGGGACGGAAACCAGGTGAGCAACGGCGGCGGCAACCTGGCGGCCACGGGTCGGTCGTCAGACCCA
AAACCCCGAGAGGGGACGGAAACATGGGATATCTGCTGCCCGCCCGGGGAGATGCTGTCCGAGGTCGTCAGACCCA
AAACCCCGAGAGGGGACGGAAACTTCGTCGACCATCATTGCCATTCCCTCTCCCCACGTGTCGTCAGACCCAAAACCC
CGAGAGGGGACGGAAACTTGCGCCAACCCTTTCGGTGTGATGCGGATGGTCGGCTCGGGTCGTCAGACCCAAAACC
CCGAGAGGGGACGGAAACCTTGAATAACGCGCAGTGAATTTCGCGGATCAGACCCAAAACCCCGAGAGGGGACGGA
AACATTCGCACGAGTTCCCGTCAGCGTCGTAAATCGCCAGTCGTCAGACCCAAAACCCCGAGAGGGGACGGAAACCC
GGCAACAATCGCGCCGGCCCGCGCGGATGACTCCGGTCGTCAGACCCAAAACCCCGAGAGGGGACGGAAACCGCA
TGGACCCGGGCGAGCTGCAGATGGTCCGGGAGGTCGTCAGACCCAAAACCCCGAGAGGGGACGGAAACTGGATTG
CGCTAACTGGCTTGGCGCTGATCCTGGTGGTCGTCAGACCCAAAACCCCGAGAGGGGACGGAAACTCCACATCGATT
TCCTTGACCTCGCCAGGAGAGAAGATCACGTCGTCAGACCCAAAACCCCGAGAGAGGACGGAAACTCGTCGACGATC
GCGTCGATGTCGATGTCCCAATCGTCGAGTCGTCAGACCCAAAACCCCGAGAGGGGACGGAAACTTGGAGCGTGTCA
CCGCAGACGGCACGATTGAGACAAGTCGTCAGACCCAAAACCCCGAGAGGGGACGGAAACCCTCAGCTCAGCATCG
CCGATGCGGTCCAGCTCGTCCGTGTCGTCAGACCCAAAACCCCGAGAGGGGACGGAAACCCAACCTCACCGCCTGC
TGGGTGAGACGTGCTCGCCGCGAGTCGTCAGACCCAAAACCCTGAACCGCCCCGGCATGTCCGGAGACTCCAGTTCT
TGGAAAGGATGGGGTCATGTCAGGTGGTTCATCGAGGAGGTACCCGCCGGAGCTGCGTGAGCGGGCGGTGCGGATG
GTCGCAGAGATCCGCGGTCAGCACGATTCGGAGTGGGCAGCGATCAGTGAGGTCGCCCGTCTACTTGGTGTTGGCTG
CGCGGAGACGGTGCGTAAGTGGGTGCGCCAGGCGCAGGTCGATGCCGGCGCACGGCCCGGGACCACGACCGAAG
AATCCGCTGAGCTGAAGCGCTTAGCGGCGGGACAACGCCGAATTGCGAAGGGCGAACGCGATTTTAAAGACCGCGT
CGGCTTTCTTCGCGGCCGAGCTCGACCGGCCAGCACGCTAATTAACGGTTCATCGCCGATCATCAGGGCCACCGCGA
GGGCCCCGATGGTTTGCGGTGGGGTGTCGAGTCGATCTGCACACAGCTGACCGAGCTGGGTGTGCCGATCGCCCCAT
CGACCTACTACGACCACATCAACCGGGAGCCCAGCCGCCGCGAGCTGCGCGATGGCGAACTCAAGGAGCACATCAG
CCGCGTCCACGCCGCCAACTACGGTGTTTACGGTGCCCGCAAAGTGTGGCTAACCCTGAACCGTGAGGGCATCGAGG
TGGCCAGATGCACCGTCGAACGGCTGATGACCAAACTCGGCCTGTCCGGGACCACCCGCGGCAAAGCCCGCAGGAC
CACGATCGCTGATCCGGCCACAGCCCGTCCCGCCGATCTCGTCCAGCGCCGCTTCGGACCACCAGCACCTAACCGGC
TGTGGGTAGCAGACCTCACCTATGTGTCGACCTGGGCAGGGTTCGCCTACGTGGCCTTTGTCACCGACGCCTACGTCG

CAGGATCCTGGGCTGGCGGGTCGCTTCCACGATGGCCACCTCCATGGTCCTCGACGCGATCGAGCAAGCCATCTGGA
CCCGCCAACAAGAAGGCGTACTCGACCTGAAAGACGTTATCCACCATACGGATAGGGGATCTCAGTACACATCGATCC
GGTTCAGCGAGCGGCTCGCCGAGGCAGGCATCCAACCGTCGGTCGGAGCGGTCGGAAGCTCCTATGACAATGCACTA
GCCGAGACGATCAACGGCCTATACAAGACCGAGCTGATCAAACCCGGCAAGCCCTGGCGGTCCATCGAGGATGTCGA
GTTGGCCACCGCGCGCTGGGTCGACTGGTTCAACCATCGCCGCCTCTACCAGTACTGCGGCGACGTCCCGCCGGTCG
AACTCGAGGCTGCCTACTACGCTCAACGCCAGAGACCAGCCGCCGGCTGAGGTCTCAGATCAGAGAGTCTCCGGACT
CACCGGGGCGGTTCACCCCGAGAGGGGACGGAAACTCGGGGAGCCGATCAGCGACCACCGCACCCTGTCAGTCGTC
AGACCCAAAACCCCGAGAGGGGACGGAAACCTTCAGCACCACCATCATCCGGCGCCTCAGCTCAGCATGTCGTCAGA
CCCAAAACCCCGAGAGGGGACGGAAACCCTTCGACGCCGGATTCGTGATCTCTTCCCGCGGATAGGTCGTCAGACCC
AAAACCCCGAGAGGGGACGGAAACTGCCCCGGCGTTTAGCGATCACAACACCAACTAATGGTCGTCAGACCCAAAAC
CCCGAGAGGGGACGGAAACCAGCGAAATACAGGCTCCACGACACGACCACAACGCGTCGTCAGACCCAAAACCCCG
AGAGGGGACGGAAACTCTTGACGATGCGGTTGCCCCGCGCCCTTTTCCAGCCGTCGTCAGACCCAAAACCCCGAGA
GGGGACGGAAACAGGTTCGCGTCAGACAGGTTCGCGTCGATCAAGTCCGGTCGTCAGACCCAAAACCCCGAGAGGG
GACGGAAACTCGGGGAGCCGATCAGCGACCACCGCACCCTGTCAGTCGTCGGACCCAAAACCCCGAGAGGGGACG
GAAACTTTATCACTCCCGACCAAATAGGTATCGGCGTGTTCAAGTCGTCAGACCCAAAACCCCGAGAGGGGACGGAAA
CTCGACACCGACATGACGGCGGTGCCGCACTTGACGCAGTCGTCAGACCCAAAACCCCGAGAGGGGACGGAAACCT
TTGCGAAGTCACCTCGCCCACACCGTCGAAGCGCCTGTCGTCAGACCCAAAACCCCGAGAGGGGACGGAAACGCGG
ATGGTGGGCAAGTTGGCGCTGGGGTCTGAGTCAAGTCGTCAGACCCAAAACCCCGAGAGGGGACGGAAACCTGCAT
CCGGAAAGTCCGTACGCTCGAAACGCTTCCAACGTGTCGTCAGACCCAAAACCCCGAGAGGGGACGGAAACTCGAA
ATCCAGCACCACATCCGCAGCTGCGGCATGCTCCCGAAGTCGTCAGACCCAAAACCCCGAGAGGGGACGGAAACGC
GAGGAACCGTCCCACCTGGGCCTGCCCCCAGCGGGTCGTCAGACCCAAAACCCCGAGAGGGGACGGAAACGCGAG
GAACCGTCCCACCTGGGCCTGCCCCCAGCGGGTCGTCAGACCCAAAACCCCGAGAGGGGACGGAAACTCAATAACA
CTTTTTTTGAGCGTGGCGCGGTTGAGAGTGTCGTCAGACCCAAAACCCCGAGAGGGGACGGAAACACGGAAACGCA
GCACCAGCCTGACAATCTTATTCTCGCGTCGTCAGACCCAAAACCCCGAGAGGGGACGGAAACATTTTGAGCGCGAA
CTCGCCCACAGTCCCCCTTTCAGGTCGTCAGACCCAAAACCCCGAGAGGGGACGGAAACGCCCCGTGGATGGCGGA
TGCGTTGTGCGCGCAAGTGTCGTCAGACCCAAAACCCCGAGAGGGGACGGAAACCCGACGATGGCCAGTAAATCGG
CGTGGGTAACCGATCCGGGTCGTCAGACCCAAAACCCCGAGAGGGGACGGAAACCCTCGCAGAAAAGGGCATCGAT
CATGAGAGTTGCGTTGATGTCGTCAGACCCAAAACCCCGAGAGGGGACGGAAACAATGCTGGCGACGATTTTCGCTG
TTGTGGTTCTCATTGTCGTCAGACCCAAAACCCCGAGAGGGGACGGAAACGCACACCAGCACCTCCCTTGACAATCC
GGCAGATCAGACGTCGTCAGACCCAAAACCCCGAGAGGGGACGGAAACTCGCGGGCTCTGGCCTAAGGGTGCTGAC
TTCGCCTGTAGTCGTCAGACCCAAAACCCCGAGAGGGGACGGAAACCCGACGACGAGCAGCGGCATACAGAGCCAC
GGATACGCCAGGTCGTCAGACCCAAAACCCCGAGAGGGGACGGAAACTTGCATCCACTCGTCGCCGACACGGCGGA
CTTCCGCGAGTCGTCAGACCCAAAACCCCGAGAGGGGACGGAAACTGGTAATTGCGTCACGGCGCGCCTGGCGGGC
CGATTGTCGTCAGACCCAAAACCCCGAGAGGGGACGGAAACACCATCCGACGCAGGCACCGAAGTCGATGACAAGC
CGTCGTCAGACCCAAAACCCCGAGAGGGGACGGAAACTGAACCGCCCCGGCATGTCCGGAGACTCCAGTTCTTGGA
AAGGATGGGGTCATGTCAGGTGGTTCATCGAGGAGGTACCCGCCGGAGCTGCGTGAGCGGGCGGTGCGGATGGTCG
CAGAGATCCGCGGTCAGCACGATTCGGAGTGGGCAGCGATCAGTGAGGTCGCCCGTCTACTTGGTGTTGGCTGCGCG
GAGACGGTGCGTAAGTGGGTGCGCCAGGCGCAGGTCGATGCCGGCGCACGGCCCGGGACCACGACCGAAGAATCC
GCTGAGCTGAAGCGCTTAGCGGCGGGACAACGCCGAATTGCGAAGGGCGAACGCGATTTTAAAGACCGCGTCGGCT
TTCTTCGCGGCCGAGCTCGACCGGCCAGCACGCTAATTAACGGTTCATCGCCGATCATCAGGGCCACCGCGAGGGCC
CCGATGGTTTGCGGTGGGGTGTCGAGTCGATCTGCACACAGCTGACCGAGCTGGGTGTGCCGATCGCCCCATCGACC
TACTACGACCACATCAACCGGGAGCCCAGCCGCCGCGAGCTGCGCGATGGCGAACTCAAGGAGCACATCAGCCGCG
TCCACGCCGCCAACTACGGTGTTTACGGTGCCCGCAAAGTGTGGCTAACCCTGAACCGTGAGGGCATCGAGGTGGCC
AGATGCACCGTCGAACGGCTGATGACCAAACTCGGCCTGTCCGGGACCACCCGCGGCAAAGCCCGCAGGACCACGA
TCGCTGATCCGGCCACAGCCCGTCCCGCCGATCTCGTCCAGCGCCGCTTCGGACCACCAGCACCTAACCGGCTGTGG
GTAGCAGACCTCACCTATGTGTCGACCTGGGCAGGGTTCGCCTACGTGGCCTTTGTCACCGACGCCTACGTCGCAGGA
TCCTGGGCTGGCGGGTCGCTTCCACGATGGCCACCTCCATGGTCCTCGACGCGATCGAGCAAGCCATCTGGACCCGC
CAACAAGAAGGCGTACTCGACCTGAAAGACGTTATCCACCATACGGATAGGGGATCTCAGTACACATCGATCCGGTTCA
GCGAGCGGCTCGCCGAGGCAGGCATCCAACCGTCGGTCGGAGCGGTCGGAAGCTCCTATGACAATGCACTAGCCGA
GACGATCAACGGCCTATACAAGACCGAGCTGATCAAACCCGGCAAGCCCTGGCGGTCCATCGAGGATGTCGAGTTGG
CCACCGCGCGCTGGGTCGACTGGTTCAACCATCGCCGCCTCTACCAGTACTGCGGCGACGTCCCGCCGGTCGAACTC
GAGGCTGCCTACTACGCTCAACGCCAGAGACCAGCCGCCGGCTGAGGTCTCAGATCAGAGAGTCTCCGGACTCACCG
GGGCGGTTCAGTCGTCAGACCCAAAACCCCGAGAGGGGACGGAAACCTGACGGCACGGAGCTTTCCGGCTTCTATC
AGGTAGTCGTCAGACCCAAAACCCCGAGAGGGGACGGAAACCCTCATGGTGGGACATGGACGAGCGCGACTATCGG
GGTCGTCAGACCCAAAACCCCGAGAGGGGACGGAAACTGGACGCAAAATCGCACCGGGTGCGGGAGGTGCAGCAG
TCGTCAGACCCAAAACCCCGAGAGGGGACGGAAACGCATATCGCCCGCCACACCACAGCCACGCTACTGCTCCATGT
CCTCAGACCCAAAACCCCGAGAGGGGACGGAAACACACCGCCGATGACAGCTATGTCCGAGTGACATCCTCCCAGTC
GTCAGACCCAAAACCACGAGAGGGGACGGAAACACACCGCCGATGACAGCTATGTCCGAGTGACATCCTCCCAGTCG
TCAGACCCAAAACCACGAGAGGGGACGGAAACTTGAACCGCCCTTCGCGCGGTGTTTCGGCCGTGCCCGAGTCGTC
AGACCCAAAACCCCGAGAGGGGACGGAAACTACGACGACTGGGTCGCCACCGCGTCTGTTGACCGGCATTCAGGAT
GATCAGTCTGCCGTGACTTCGGCGATGGCGG

CRISPR annotation :
['starting_pattern1', 'DR0', 'esp1', 'DR0', 'esp2', 'DR0', 'esp3', 'DR0', 'esp4', 'DR0', 'esp5', 'DR0', 'esp6', 'DR0', 'esp7', 'DR0', 'esp8', 'DR0', 'esp9', 'DR0', 'esp10', 'DR0', 'esp11(var)', 'DR0', 'esp12', 'DR0', 'esp13', 'DR0', 'esp14', 'DR0', 'esp15', 'DR0', 'esp16', 'DR0', 'esp17', 'DR0', 'esp18', 'DR0', 'esp19', 'DR0', 'esp20', 'DR0', 'esp21', 'DR0', 'esp22', 'DR0', 'esp23', 'DR0', 'esp24', 'DR0', 'esp25', 'DRb2', 'esp26', 'DR0', 'esp27', 'DR0', 'esp28', 'DR0', 'esp29', 'DR0', 'esp30', 'DR2', 'esp31', 'DR0', 'esp32', 'DR0', 'esp33(var)', 'DR0', 'esp34', 'rDRa1', 'IS6110', 'DRb1', 'esp35', 'DR0', 'esp36', 'DR0', 'esp37', 'DR0', 'esp38', 'DR0', 'esp39', 'DR0', 'esp40', 'DR0', 'esp41', 'DR0', 'esp35', 'DR0', 'esp42', 'DR0', 'esp43', 'DR0', 'esp44', 'DR0',

'esp45', 'DR0', 'esp46', 'DR0', 'esp47', 'DR0', 'esp48', 'DR0', 'esp48', 'DR0', 'esp49', 'DR0', 'esp50', 'DR0', 'esp51(var)', 'DR0', 'esp52', 'DR0', 'esp53', 'DR0', 'esp54', 'DR0', 'esp55', 'DR0', 'esp56', 'DR0', 'esp57', 'DR0', 'esp58', 'DR0', 'esp59', 'DR0', 'esp60', 'DR0', 'esp61', 'DR0', 'IS6110', 'DR0', 'esp63', 'DR0', 'esp64', 'DR0', 'esp65(var)', 'DR0', 'esp66', 'DR4', 'esp67', 'DR5', 'esp67', 'DR5', 'esp68', 'DR0', 'ending_pattern1', 'Rv2813c']

=> two tandem duplications and a second IS, and 4 spacer variants.

A.II. CRASS results

1. CRISPR detection

Command :
./crass SRA_75_shuffled.fasta

Result :
[crass_patternFinder]: Processed 5988 ...0 sec
[crass_clusterCore]: 0 variants mapped to 0 clusters
[crass_clusterCore]: creating non-redundant set[crass_patternFinder]: Found 0 reads
[crass_graphBuilder]: 0 CRISPRs found!

Interpretation:
No CRISPR (DR and spacers) found.

A.III. CRISPR_detector

Command :
crispr Finder SRA_75_shuffled.fasta 23 45

Results :
Number of frequent kmers is 334 167
Number of relevant reads is 1124
GTCGTCAGACCCAAAACCCCGAGAGGGGACGGAAAC GTTTCCGTCCCCTCTCGGGGTTTTGGGTCTGACGAC
STATS
Reads: 0.00537014007568
Stats: 0.178255081177
Refined Stats: 0.0217080116272
Refined Reads: 0.0207557678223
Update Kmers: 0.0186409950256
Duplication: 0.00965785980225
Hash Computation: 0.502161026001
Match Kmers: 0.0011579990387
Good Analysis : 0.684767961502
Bad Analysis: 0.151758432388
Clustering: 0.00128507614136
Canonization: 0.919391155243
Alignment: 0.137055158615
Total 2.66886901855

Interpretation:
The CRISPR has been detected, but the unique result is the DR0 sequence (no information about spacer, structure, etc.)

A.IV. CRISPRbuilder-TB

Produced (automatic) results :

('***starting_pattern1***DR0*esp1*DR0*esp2*DR0*esp3[:11]', 1890)
('esp2[31:]*DR0*esp3*DR0*esp4[:11]', 675)
('esp3[26:]*DR0*esp4*DR0*esp5*DR0*esp6*DR0*esp7*DR0*esp8*DR0*esp9*DR0*esp10*DR0*esp11[:10]', 5310)
('esp10[30:]*DR0*TTGAACACGGAGCCTTGCACATGCCGTGGCTCAGGGGT*DR0*esp12[:8]', 630)
('esp11[25:]*DR0*esp12*DR0*esp13*DR0*esp14[:10]', 1440)
('esp13[24:]*DR0*esp14*DR0*esp15*DR0*esp16[:11]', 1440)
('esp15[28:]*DR0*esp16*DR0*esp17*DR0*esp18*DR0*esp19[:11]', 2295)
('esp18[27:]*DR0*esp19*DR0*esp20*DR0*esp21[:11]', 1395)

('esp20[27:]*DR0*esp21*DR0*esp22[:10]', 720)
('esp21[30:]*DR0*esp22*DR0*esp23[:10]', 720)
('esp22[29:]*DR0*esp23*DR0*esp24[:10]', 675)
('esp23[25:]*DR0*esp24*DR0*esp25*DRb2*esp26*DR0*esp27*DR0*esp28[:11]', 2880)
('esp27[27:]*DR0*esp28*DR0*esp29[:11]', 675)
('esp28[26:]*DR0*esp29*DR0*esp30[:11]', 675)
('esp29[26:]*DR0*esp30*DR2*esp31*DR0*esp32*DR0**CCTCAGCTCAGCATCGCCGATGCGGTCCAGCTCGTCCGT**DR0*esp34[:10]', 3180)
('esp33[28:]*DR0*esp34*rDRa1***IS6110deb***GGTCATGTCAGGTGGT', 1125)
('GACCAGCCGCCGGCTGAGGTCTC***finIS6110*DRb1***esp35*DR0[:6]', 720)
('DR0[19:]*esp35*DR0*esp36*DR0*esp37*DR0*esp38[:10]', 1962)
('esp37[27:]*DR0*esp38*DR0*esp39[:10]', 675)
('esp38[25:]*DR0*esp39*DR0*esp40[:10]', 675)
('esp39[25:]*DR0*esp40*DR0*esp41*DR0*esp35*GTC', 1704)
('DR0[19:]*esp35*DR6*esp42*DR0*esp43*DR0*esp44*DR0*esp45*DR0*esp46*DR0*esp47*DR0*esp48*DR0*', 6588)
('esp48[14:]***DR0*esp48**[:10]', 54)
('esp48[25:]*DR0*esp48[:23]', 72)
('esp48[14:]*DR0*esp49[:10]', 54)
('esp48[29:]*DR0*esp49*DR0*esp50*DR0**ATTTTGAGCGCGAACTCGCCCACAGTCCCCCTTTCAG**DR0*esp52*DR0*esp53*DR0*esp54*DR0*esp55*DR0*esp56*DR0*esp57*DR0*esp58*DR0*esp59*DR0*esp60*DR0***esp61*DR0*IS6110[:21]'**, 10650)
('GACCAGCCGCCGGCTGAGGTCTC***finIS6110***DR0*esp63*DR0*esp64*DR0**TGGACGCAAAATCGCACCGGGTGCGGGAGGTGCAGCA**DR0*esp66*DR4*esp67*DR5*', 4926)
('esp67[18:]***DR5*esp67***GT', 474)
('esp67[18:]*DR5*esp68*DR0*ending_pattern1*Rv2813c[:25]', 1593)

('TCCGGCGGGTACCTCCTCGATGAACCACCTGACATGACCCCATCCTTTCC*finIS6110c*GTTTCCGTCCCCTCTCGGGGTTTTGGGTCTGACGACGGCTTGTCATCG', 927)
('TGACATGACCCCATCCTTTCC*finIS6110c*esp81[19:24]', 12)
('GGCTGCCTACTACGCTCAACGCCAGAGACCAGCCGCCGGCTGAGGTCTC*finIS6110*', 438)
('CATGTCCGGAGACTCCAGTTCTTGGAAAGGATGGGGTCATGTCAGGTGGTTCATCGAGGAGGTACCCGCCGGAGCTGCGTGA', 225)
('GTGAGTCCGGAGACTCTCTGATCTGAGACCTCAGCCGGCGGCTGGTCTCTGGCGTTGAGCGTAGTAGGCAGCCTCGAGTTCG', 225)

Manual investigation :

Several contigs are produced, due to the small read length. However, they overlap well, and the last contigs indicate putative tandem duplications of spacers 48 and 67. Investigating reads with not consecutive spacers (SRA_75.not_consecutive file) provides :

(('esp35', 'esp42(3)'), 12)
(('esp41', 'esp35(1)'), 6)
(('esp48', 'esp48'), 3)
(('esp67', 'esp67'), 12)

which reinforces us in this option, leading to subsequent CRISPR structure after manual inspection :

*starting_pattern1*DR0*esp1*DR0*esp2*DR0*esp3*DR0*esp4*DR0*esp5*DR0*esp6*DR0*esp7*DR0*esp8*DR0*esp9*DR0*esp10*DR0***esp11(var)***DR0*esp12*DR0*esp13*DR0*esp14*DR0*esp15*DR0*esp16*DR0*esp17*DR0*esp18*DR0*esp19*DR0*esp20*DR0*esp21*DR0*esp22*DR0*esp23*DR0*esp24*DR0*esp25*DRb2*esp26*DR0*esp27*DR0*esp28*DR0*esp29*DR0*esp30*DR2*esp31*DR0*esp32*DR0***esp33(var)***DR0*esp34*rDRa1*IS6110*DRb1*esp35*DR0*esp36*DR0*esp37*DR0*esp38*DR0*esp39*DR0*esp40*DR0*esp41*DR0*esp35*DR6*esp42*DR0*esp43*DR0*esp44*DR0*esp45*DR0*esp46*DR0*esp47*DR0*esp48*DR0***esp48*DR0***esp49*DR0*esp50*DR0***esp51(var)***DR0*esp52*DR0*esp53*DR0*esp54*DR0*esp55*DR0*esp56*DR0*esp57*DR0*esp58*DR0*esp59*DR0*esp60*DR0*esp61*DR0***IS6110*DR0***esp63*DR0*esp64*DR0***esp65(var)***DR0*esp66*DR4*esp67*DR5***esp67*DR5***esp68*DR0*ending_pattern1*Rv2813c[:25]

Interpretation:
Full locus recovery with perfect identification of spacer variants, duplications and IS*6110* insertion after manual reconstruction.

B- Simulation and reconstruction of CRISPR, CRISPR-5

B.I. Simulated data

Evolution characteristics :
0.5% mutation.
Tandem duplications allowed.
IS6110 insertions allowed.

Length reads : 125.

Nucleotidic sequence :
CATCATCAGCAGGCATTGTTACCACACGCTGGACGAATTGTCCATAGAGTCGTCAGACCCAAAACCCCGAGAGGGGACGGAAACTTA
AAACCGTGTTGCACTGCAACCCGGAATTCTTGCACGTCGTCAGACCCAAAACCCCGAGAGGGGACGGAAACCATAGAGGGTCGCC
GGCTCTGGATCACGCTCCCCTAGTCGTGTCGTCAGACCCAAAACCCCGAGAGGGGACGGAAACTTTTTGCCTCATGCTTGGGCGA
CAGCTTTTGACCAAGTCGTCAGACCCAAAACCCCGAGAGGGGACGGAAACTCGCAAGCGCCGTGCTTCCAGTGATCGCCTTCTAGT
CGTCAGACCCAAAACCCCGAGAGGGGACGGAAACTCGCGGCGCGGCATGGCACGGCAGGCGTGGCTAGGGGGTCGTCAGACCC
AAAACCCCGAGAGGGGACGGAAACATGTGCGCCGTCGCCGTAAGTGCCCCACGGCCCGTGTCGTCAGACCCAAAACCCCGAGAG
GGGACGGAAACATTTCGACGACAATTCGTTGACCACGGATTTTCAGAGTCGTCAGACCCAAAACCCCGAGAGGGGACGGAAACACA
TCCCACGCGTTACCGCTGGCGCGCATCATTCATCGAGTCGTCAGACCCAAAACCCCGAGAGGGGACGGAAACCCATATCGGGGAC
GGCGACGCTGCGAGAGGACACGCCGAGTCGTCAGACCCAAAACCCCGAGAGGGGACGGAAACTACACCACGCGTCGTGCCATCA
GTCAGCGTCCTCCTCGTCGTCAGACCCAAAACCCCGAGAGGGGACGGAAACTTGAACACGGAGCCGTGCACATGCCGTGGCTCA
GGGGTGTCGTCAGACCCAAAACCCCGAGAGGGGACGGAAACAACACCTCAGTAGCACGTCATACGCCGACCAATCATCAGGTCGT
CAGACCCAAAACCCCGAGAGGGGACGGAAACTTTTCTGACCACTTGTGCGGGATTAGCGGGCTTAGGTCGTCAGACCCAAAACCC
CGAGAGGGGACGGCAACACCAATGCGTCGTCATTTCCGGCTTCAATTTCAGCCTGTCGTCAGACCCAAAACCCCGAGAGGGGACG
GAAACCTGAGGAGAGCGAGTACTCGGGGCTGCCGTCTGCGCTGGTCGTCAGACCCAAAACCCCGAGAGGGGACGGAAACACGAC
GTTAGGGCATGCAGCATGCCGTCCCCGTTTTTGAGTCGTCAGACCCAAAACCCCGAGAGGGGACGGAAACTGCTCTTGAGCAACG
CCATCATCCGGCGCCGCAGCTCCGCGTCGTCAGACCCAAAACCCCGAGAGGGGACGGAAACGCGTGAAACCGCCCCCAGCCTCG
CCGGGGCCGCCTAGGTCGTCAGACCCAAAACCCCGAGAGGGGACGGAAACACTCGGAATCCCATGTGCTGACAGCGGATTCGCAT
GTCGTCAGACCCAAAACCCCGAGAGGGGACGGAAACCGGGCAGCGTTCGACACCCGCTCTAGTTGACTTCCGGGTCGTCAGACC
CAAAACCCCGAGAGGGGACGGAAACCAGGTGAGCAACGGCGGCGGCAACCTGGCGGCCACGGGTCGGTCGTCAGACCCAAAAC
CCCGAGAGGGGACGGAAACATGGGATATCTGCTGCCCGCCCGGGGAGATGCTGTCCGAGGTCGTCAGACCCAAAACCCCGAGAG
GGGACGGAAACTTCGTCGACCATCATTGCCATTCCCTCTCCCCACGTGTCGTCAGACCCAAAACCCCGAGAGGGGACGGAAACTTG
CGCCAACCCTTTCGGTGTGATGCGGATGGTCGGCTCGGGTCGTCAGACCCAAAACCCCGAGAGGGGACGGAAACCTTGAATAACG
CGCAGTGAATTTCGCGGATCAGACCCAAAACCCCGAGAGGGGACGGAAACTGAACCGCCCCGGCATGTCCGGAGACTCCAGTTCT
TGGAAAGGATGGGGTCATGTCAGGTGGTTCATCGAGGAGGTACCCGCCGGAGCTGCGTGAGCGGGCGGTGCGGATGGTCGCAGA
GATCCGCGGTCAGCACGATTCGGAGTGGGCAGCGATCAGTGAGGTCGCCCGTCTACTTGGTGTTGGCTGCGCGGAGACGGTGCGT
AAGTGGGTGCGCCAGGCGCAGGTCGATGCCGGCGCACGGCCCGGGACCACGACCGAAGAATCCGCTGAGCTGAAGCGCTTAGC
GGCGGGACAACGCCGAATTGCGAAGGGCGAACGCGATTTTAAAGACCGCGTCGGCTTTCTTCGCGGCCGAGCTCGACCGGCCAG
CACGCTAATTAACGGTTCATCGCCGATCATCAGGGCCACCGCGAGGGCCCCGATGGTTTGCGGTGGGGTGTCGAGTCGATCTGCAC
ACAGCTGACCGAGCTGGGTGTGCCGATCGCCCCATCGACCTACTACGACCACATCAACCGGGAGCCCAGCCGCCGCGAGCTGCG
CGATGGCGAACTCAAGGAGCACATCAGCCGCGTCCACGCCGCCAACTACGGTGTTTACGGTGCCCGCAAAGTGTGGCTAACCCTG
AACCGTGAGGGCATCGAGGTGGCCAGATGCACCGTCGAACGGCTGATGACCAAACTCGGCCTGTCCGGGACCACCCGCGGCAAA
GCCCGCAGGACCACGATCGCTGATCCGGCCACAGCCCGTCCCGCCGATCTCGTCCAGCGCCGCTTCGGACCACCAGCACCTAAC
CGGCTGTGGGTAGCAGACCTCACCTATGTGTCGACCTGGGCAGGGTTCGCCTACGTGGCCTTTGTCACCGACGCCTACGTCGCAG
GATCCTGGGCTGGCGGGTCGCTTCCACGATGGCCACCTCCATGGTCCTCGACGCGATCGAGCGAGCCATCTGGACCCGCCAACAA
GAAGGCGTACTCGACCTGAAAGACGTTATCCACCATACGGATAGGGGATCTCAGTACACATCGATCCGGTTCAGCGAGCGGCTCGC
CGAGGCAGGCATCCAACCGTCGGTCGGAGCGGTCGGAAGCTCCTATGACAATGCACTAGCCGAGACGATCAACGGCCTATACAAGA
CCGAGCTGATCAAACCCGGCAAGCCCTGGCGGTCCATCGAGGATGTCGAGTTGGCCACCGCGCGCTGGGTCGACTGGTTCAACCA
TCGCCGCCTCTACCAGTACTGCGGCGACGTCCCGCCGGTCGAACTCGAGGCTGCCTACTACGCTCAACGCCAGAGACCAGCCGCC
GGCTGAGGTCTCAGATCAGAGAGTCTCCGGACTCACCGGGGCGGTTCAGTCGTCAGACCCAAAACCCCGAGAGGGGACGGAAAC
CCGGCAACAATCGCGCCGGCCCGCGCGGATGACTCCGGTCGTCAGACCCAAAACCCCGAGAGGGGACGGAAACCGCATGGACCC
GGGCGAGCTGCAGATGGTCCGGGAGGTCGTCAGACCCAAAACCCCGAGAGGGGACGGAAACTGGATTGCGCTAACTGGCTTGGC
GCTGATCCTGGTGGTCGTCAGACCCAAAACCCCGAGAGGGGACGGAAACTCCACATCGATTTCCTTGACCTCGCCAGGAGAGAAG
ATCACGTCGTCAGACCCAAAACCCCGAGAGAGGACGGAAACTCGTCGACGATCGCGTCGATGTCGATGTCCCAATCGTCGAGTCGT
CAGACCCAAAACCCCGAGAGGGGACGGAAACTTGGAGCGTGTCACCGCAGACGGCACGATTGAGACAAGTCGTCAGACCCAAAAC
CCCGAGAGGGGACGGAAACCCTCAGCTCAGCATCGCTGATGCGGTCCAGCTCGTCCGTGTCGTCAGACCCAAAACCCCGAGAGG
GGACGGAAACCCAACCTCACCGCCTGCTGGGTGAGACGTGCTCGCCGCGAGTCGTCAGACCCAAAACCCTGAACCGCCCCGGCA
TGTCCGGAGACTCCAGTTCTTGGAAAGGATGGGGTCATGTCAGGTGGTTCATCGAGGAGGTACCCGCCGGAGCTGCGTGAGCGGG
CGGTGCGGATGGTCGCAGAGATCCGCGGTCAGCACGATTCGGAGTGGGCAGCGATCAGTGAGGTCGCCCGTCTACTTGGTGTTGG
CTGCGCGGAGACGGTGCGTAAGTGGGTGCGCCAGGCGCAGGTCGATGCCGGCGCACGGCCCGGGACCACGACCGAAGAATCCG
CTGAGCTGAAGCGCTTAGCGGCGGGACAACGCCGAATTGCGAAGGGCGAACGCGATTTTAAAGACCGCGTCGGCTTTCTTCGCGG
CCGAGCTCGACCGGCCAGCACGCTAATTAACGGTTCATCGCCGATCATCAGGGCCACCGCGAGGGCCCCGATGGTTTGCGGTGGG
GTGTCGAGTCGATCTGCACACAGCTGACCGAGCTGGGTGTGCCGATCGCCCCATCGACCTACTACGACCACATCAACCGGGAGCC
CAGCCGCCGCGAGCTGCGCGATGGCGAACTCAAGGAGCACATCAGCCGCGTCCACGCCGCCAACTACGGTGTTTACGGTGCCCG
CAAAGTGTGGCTAACCCTGAACCGTGAGGGCATCGAGGTGGCCAGATGCACCGTCGAACGGCTGATGACCAAACTCGGCCTGTCC
GGGACCACCCGCGGCAAAGCCCGCAGGACCACGATCGCTGATCCGGCCACAGCCCGTCCCGCCGATCTCGTCCAGCGCCGCTTC
GGACCACCAGCACCTAACCGGCTGTGGGTAGCAGACCTCACCTATGTGTCGACCTGGGCAGGGTTCGCCTACGTGGCCTTTGTCA
CCGACGCCTACGTCGCAGGATCCTGGGCTGGCGGGTCGCTTCCACGATGGCCACCTCCATGGTCCTCGACGCGATCGAGCAAGCC
ATCTGGACCCGCCAACAAGAAGGCGTACTCGACCTGAAAGACGTTATCCACCATACGGATAGGGGATCTCAGTACACATCGATCCGG
TTCAGCGAGCGGCTCGCCGAGGCAGGCATCCAACCGTCGGTCGGAGCGGTCGGAAGCTCCTATGACAATGCACTAGCCGAGACGA
TCAACGGCCTATACAAGACCGAGCTGATCAAACCCGGCAAGCCCTGGCGGTCCATCGAGGATGTCGAGTTGGCCACCGCGCGCTG
GGTCGACTGGTTCAACCATCGCCGCCTCTACCAGTACTGCGGCGACGTCCCGCCGGTCGAACTCGAGGCTGCCTACTACGCTCAA

CGCCAGAGACCAGCCGCCGGCTGAGGTCTCAGATCAGAGAGTCTCCGGACTCACCGGGGCGGTTCACCCCGAGAGGGGACGGA
AACTCGGGGAGCCGATCAGCGACCACCGCACCCTGTCAGTCGTCAGACCCAAAACCCCGAGAGGGGACGGAAACCTTCAGCACC
ACCATCATCCGGCGCCTCAGCTCAGCATGTCGTCAGACCCAAAACCCCGAGAGGGGACGGAAACCCTTCGACGCCGGATTCGTGA
TCTCTTCCCGCGGATAGGTCGTCAGACCCAAAACCCCGAGAGGGGACGGAAACTGCCCCGGCGTTTAGCGATCACAACACCAACTA
ATGGTCGTCAGACCCAAAACCCCGAGAGGGGACGGAAACCAGCGAAATACAGGCTCCACGACACGACCACAACGCGTCGTCAGAC
CCAAAACCCCGAGAGGGGACGGAAACTCTTGACGATGCGGTTGCCCCGCGCCCTTTTCCAGCCGTCGTCAGACCCAAAACCCCGA
GAGGGGACGGAAACAGGTTCGCGTCAGACAGGTTCGCGTCGATCAAGTCCGGTCGTCAGACCCAAAACCCCGAGAGGGGACGGA
AACTCGGGGAGCCGATCAGCGACCACCGCACCCTGTCAGTCGTCAGACCCAAAACCCCGAGAGGGGACGGAAACTTTATCACTCC
CGACCAAATAGGTATCGGCGTGTTCAAGTCGTCAGACCCAAAACCCCGAGAGGGGACGGAAACTCGACACCGACATGACGGCGGT
GCCGCACTTGACGCAGTCGTCAGACCCAAAACCCCGAGAGGGGACGGAAACCTTTGCGAAGTCACCTCGCCCACACCGTCGAAG
CGCCTGTCGTCAGACCCAAAACCCCGAGAGGGGACGGAAACGCGGATGGTGGGCAAGTTGGCGCTGGGGTCTGAGTCAAGTCGT
CAGACCCAAAACCCCGAGAGGGGACGGAAACCTGCATCCGGAAAGTCCGTACGCTCGAAACGCTTCCAACGTGTCGTCAGACCCA
AAACCCCGAGAGGGGACGGAAACTCGAAATCCAGCACCACATCCGCAGCTGCGGCATGCTCCCGAAGTCGTCAGACCCAAAACCC
CGAGAGGGGACGGAAACGCGAGGAACCGTCCCACCTGGGCCTGCCCCCAGCGGGTCGTCAGACCCAAAACCCCGAGAGGGGAC
GGAAACTCAATAACACTTTTTTTGAGCGTGGCGCGGTTGAGAGTGTCGTCAGACCCAAAACCCCGAGAGGGGACGGAAACACGGA
AACGCAGCACCAGCCTGACAATCTTATTCTCGCGTCGTCAGACTCAAAACCCCGAGAGGGGACGGAAACATTTTGAGCGCGAACTC
GTCCACAGTCCCCCTTTCAGGTCGTCAGACCCAAAACCCCGAGAGGGGACGGAAACGCCCCGTGGATGGCGGATGCGTTTTGCGC
GCAAGTGTCGTCAGACCCAAAACCCCGAGAGGGGACGGAAACCCGACGATGGCCAGTAAATCGGCGTGGGTAACCGATCCGGGTC
GTCAGACCCAAAACCCCGAGAGGGGACGGAAACCCTCGCAGAAAAGGGCATCGATCATGAGAGTTGCGTTGATGTCGTCAGACCC
AAAACCCCGAGAGGGGACGGAAACAATGCTGGCGACGATTTTCGCTGTTGTGGTTCTCATTTGAACCGCCCCGGCATGTCCGGAGA
CTCCAGTTCTTGGAAAGGATGGGGTCATGTCAGGTGGTTCATCGAGGAGGTACCCGCCGGAGCTGCGTGAGCGGGCGGTGCGGAT
GGTCGCAGAGATCCGCGGTCAGCACGATTCGGAGTGGGCAGCGATCAGTGAGGTCGCCCGTCTACTTGGTGTTGGCTGCGCGGA
GACGGTGCGTAAGTGGGTGCGCCAGGCGCAGGTCGATGCCGGCGCACGGCCCGGGACCACGACCGAAGAATCCGCTGAGCTGA
AGCGCTTAGCGGCGGGACAACGCCGAATTGCGAAGGGCGAACGCGATTTTAAAGACCGCGTCGGCTTTCTTCGCGGCCGAGCTCG
ACCGGCCAGCACGCTAATTAACGGTTCATCGCCGATCATCAGGGCCACCGCGAGGGCCCCGATGGTTTGCGGTGGGGTGTCGAGT
CGATCTGCACACAGCTGACCGAGCTGGGTGTGCCGATCGCCCCATCGACCTACTACGACCACATCAACCGGGAGCCCAGCCGCCG
CGAGCTGCGCGATGGCGAACTCAAGGAGCACATCAGCCGCGTCCACGCCGCCAACTACGGTGTTTACGGTGCCCGCAAAGTGTGG
CTAACCCTGAACCGTGAGGGCATCGAGGTGGCCAGATGCACCGTCGAACGGCTGATGACCAAACTCGGCCTGTCCGGGACCACCC
GCGGCAAAGCCCGCAGGACCACGATCGCTGATCCGGCCACAGCCCGTCCCGCCGATCTCGTCCAGCGCCGCTTCGGACCACCAG
CACCTAACCGGCTGTGGGTAGCAGACCTCACCTATGTGTCGACCTGGGCAGGGTTCGCCTACGTGGCCTTTGTCACCGACGCCTAC
GTCGCAGGATCCTGGGCTGGCGGGTCGCTTCCACGATGGCCACCTCCATGGTCCTCGACGCGATCGAGCAAGCCATCTGGACCCG
CCAACAAGAAGGCGTACTCGACCTGAAAGACGTTATCCACCATACGGATAGGGGATCTCAGTACACATCGATCCGGTTCAGCGAGCG
GCTCGCCGAGGCAGGCATCCAACCGTCGGTCGGAGCGGTCGGAAGCTCCTATGACAATGCACTAGCCGAGACGATCAACGGCCTA
TACAAGACCGAGCTGATCAAACCCGGCAAGCCCTGGCGGTCCATCGAGGATGTCGAGTTGGCCACCGCGCGCTGGGTCGACTGGT
TCAACCATCGCCGCCTCTACCAGTACTGCGGCGACGTCCCGCCGGTCGAACTCGAGGCTGCCTACTACGCTCAACGCCAGAGACC
AGCCGCCGGCTGAGGTCTCAGATCAGAGAGTCTCCGGACTCACCGGGGCGGTTCAGCACACCAGCACCTCCCTTGACAATCCGGC
AGATCAGACGTCGTCAGACCCAAAACCCCGAGAGGGGACGGAAACTCGCGGGCTCTGGCCTAAGGGTGCTGACTTCGCCTGTAGT
CGTCAGACCCAAAACCCCGAGAGGGGACGGAAACCCGACGACGAGCAGCGGCATACAGAGCCACGGATACGCCAGGTCGTCAGA
CCCAAAACCCCGAGAGGGGACGGAAACTTGCATCCACTCGTCGCCGACACGGCGGACTTCCGCGAGTCGTCAGACCCAAAACCC
CGAGAGGGGACGGAAACTGGTAATTGCGTCACGGCGCGCCTGGCGGGCCGATTGTCGTCAGACCCAAAACCCCGAGAGGGGACG
GAAACACCATCCGACGCAGGCACCGAAGTCGATGACAAGCCGTCGTCAGACCCAAAACCCCGAGAGGGGACGGAAACTAGTACGC
CATCTGTGCCTCATACAGGTCCAGTGCCCTGTCGTCAGACCCAAAACCCCGAGAGGGGACGGAAACCTGACGGCACGGAGCTTTC
CGGCTTCTATCAGGTAGTCGTCAGACCCAAAACCCCGAGAGGGGACGGAAACCCTCATGGTGGGACATGGACGAGCGCGACTATC
GGGGTCGTCAGACCCAAAACCCCGAGAGGGGACGGAAACTGGACGCAGAATCGCACCGGGTGCGGGAGGTGCAGCAGTCGTCA
GACCCAAAACCCCGAGAGGGGACGGAAACGCATATCGCCCGCCACACCACAGCCACGCTACTGCTCCATGTCCTCAGACCCAAAA
CCCCGAGAGGGGACGGAAACACACCGCCGATGACAGCTATGTCCGAGTGACATCCTCCCAGTCGTCAGACCCAAAACCACGAGAG
GGGACGGAAACTTGAACCGCCCTTCGCGCGGTGTTTCGGCCGTGCCCGAGTCGTCAGACCCAAAACCCCGAGAGGGGACGGAAA
CTACGACGACTGGGTCGCCACCGCGTCTGTTGACCGGCATTCAGGATGATCAGTCTGCCGTGACTTCGGCGATGGCGG

CRISPR annotation :
*starting_pattern1*DR0*esp1*DR0*esp2*DR0*esp3*DR0*esp4*DR0*esp5*DR0*esp6*DR0*esp7(var)*DR0*esp8*DR0*esp9*DR0*esp10*DR0*esp11*DR0*esp12*DR0*esp13*DRv*esp14*DR0*esp15*DR0*esp16*DR0*esp17*DR0*esp18*DR0*esp19*DR0*esp20*DR0*esp21*DR0*esp22*DR0*esp23*DR0*esp24*DR0*esp25***DRb2*IS6110***DR0*esp27*DR0*esp28*DR0*esp29*DR0*esp30*DR2*esp31*DR0*esp32*DR0*esp33*DR0*esp34*rDRa1*IS6110*DRb1*esp35*DR0*esp36*DR0*esp37*DR0*esp38*DR0*esp39*DR0*esp40*DR0*esp41*DR0*esp35*DR0*esp42*DR0*esp43*DR0*esp44*DR0*esp45*DR0*esp46*DR0*esp47*DR0*esp48*DR0*esp49*DR0*esp50*DR25*esp51*DR0*esp52(var)*DR0*esp53*DR0*esp54*DR0*esp55*IS6110*esp56*DR0*esp57*DR0*esp58*DR0*esp59*DR0*esp60*DR0*esp61*DR0*esp62*DR0*esp63*DR0*esp64*DR0*esp65*DR0*esp66*DR4*esp67*DR5*esp68*DR0*ending_pattern1*Rv2813c

=> no tandem duplications, 1 IS insertion, 2 DR variants and 2 spacer variants.

B.II. CRASS results

1. CRISPR detection

Command :
./crass SRA_125_shuffled.fasta

Result :
1 CRISPRs found

2. DR investigation

Command :
crisprtools extract -d crass.crispr

Result :
GTCGTCAGACCCAAAACCCCGAGAGGGGACGGAAAC

Interpretation – part 1:
DR0 has been recovered well, but no variant found.

3. Spacer investigation

Command :
crisprtools extract -s crass.crispr

Result :
>G1SP6_Cov_57
TTAAAACCGTGTTGCACTGCAACCCGGAATTCTTGCAC => spacer 1
>G1SP1750_Cov_24
TTGAACCGCCCTTCGCGCGGTGTTTCGGCCGTGCCCGA => spacer 68
>G1SP1244_Cov_60
TCGCAAGCGCCGTGCTTCCAGTGATCGCCTTCTA => spacer 4
>G1SP13_Cov_54
TCGCGGCGCGGCATGGCACGGCAGGCGTGGCTAGGGG => spacer 5
>G1SP443_Cov_54
TCAATAACACTTTTTTTGAGCGTGGCGCGGTTGAGAGT => spacer 49
>G1SP458_Cov_54
ACGGAAACGCAGCACCAGCCTGACAATCTTATTCTCGC => spacer 50
>G1SP19_Cov_60
ATGTGCGCCGTCGCCGTAAGTGCCCCACGGCCCGT => spacer 6
>G1SP25_Cov_60
ATTTCGACGACAATTCGTTGACCACGGATTTTCAGA => spacer 7
>G1SP970_Cov_57
TACACCACGCGTCGTGCCATCAGTCAGCGTCCTCCTC => spacer 10
>G1SP32_Cov_57
TTGAACACGGAGCCGTGCACATGCCGTGGCTCAGGGGT => spacer 11
>G1SP2003_Cov_60
TTTTCTGACCACTTGTGCGGGATTAGCGGGCTTAG => spacer 13
>G1SP39_Cov_42
ACCAATGCGTCGTCATTTCCGGCTTCAATTTCAGCCT => spacer 14
>G1SP895_Cov_57
GCGTGAAACCGCCCCCAGCCTCGCCGGGGCCGCCTAG => spacer 18
>G1SP1030_Cov_57
AGGTTCGCGTCAGACAGGTTCGCGTCGATCAAGTCCG => spacer 41
>G1SP46_Cov_60
ACTCGGAATCCCATGTGCTGACAGCGGATTCGCAT => spacer 19
>G1SP491_Cov_51
CCGACGATGGCCAGTAAATCGGCGTGGGTAACCGATCCGG => spacer 53
>G1SP53_Cov_57
CCGGCAACAATCGCGCCGGCCCGCGCGGATGACTCCG => spacer 27
>G1SP352_Cov_54
CTTCAGCACCACCATCATCCGGCGCCTCAGCTCAGCAT => spacer 36
>G1SP60_Cov_57
CCTTCGACGCCGGATTCGTGATCTCTTCCCGCGGATAG => spacer 37
>G1SP920_Cov_57
TGGACGCAGAATCGCACCGGGTGCGGGAGGTGCAGCA => spacer 65
>G1SP2132_Cov_60
TGCCCCGGCGTTTAGCGATCACAACACCAACTAATG => spacer 38
>G1SP67_Cov_54
CAGCGAAATACAGGCTCCACGACACGACCACAACGC => spacer 39
>G1SP375_Cov_57
CTTTGCGAAGTCACCTCGCCCACACCGTCGAAGCGCCT => spacer 44
>G1SP74_Cov_57
GCGGATGGTGGGCAAGTTGGCGCTGGGGTCTGAGTCAA => spacer 45
>G1SP509_Cov_51
CCTCGCAGAAAAGGGCATCGATCATGAGAGTTGCGTTGAT => spacer 54
>G1SP422_Cov_48
TCGAAATCCAGCACCACATCCGCAGCTGCGGCATGCTCCCGAA => spacer 47

>G1SP81_Cov_54
GCGAGGAACCGTCCCACCTGGGCCTGCCCCCAGCGG => spacer 48
>G1SP2145_Cov_57
ATTTTGAGCGCGAACTCGTCCACAGTCCCCCTTTCAG => spacer 51
>G1SP88_Cov_60
GCCCCGTGGATGGCGGATGCGTTTTGCGCGCAAGT => spacer 52
>G1SP95_Cov_54
TCGCGGGCTCTGGCCTAAGGGTGCTGACTTCGCCTGTA => spacer 57
>G1SP911_Cov_48
CCGACGACGAGCAGCGGCATACAGAGCCACGGATACGCCAG => spacer 58
>G1SP102_Cov_57
TTGCATCCACTCGTCGCCGACACGGCGGACTTCCGCGA => spacer 59
>G1SP965_Cov_51
CCATATCGGGGACGGCGACGCTGCGAGAGGACACGCCGA => spacer 9
>G1SP108_Cov_60
TGGTAATTGCGTCACGGCGCGCCTGGCGGGCCGATT => spacer 60
>G1SP114_Cov_57
ACCATCCGACGCAGGCACCGAAGTCGATGACAAGCC => spacer 61
>G1SP578_Cov_57
TAGTACGCCATCTGTGCCTCATACAGGTCCAGTGCCCT => spacer 62
>G1SP121_Cov_60
CTGACGGCACGGAGCTTTCCGGCTTCTATCAGGTA => spacer 63
>G1SP127_Cov_54
CCTCATGGTGGGACATGGACGAGCGCGACTATCGGG => spacer 64
>G1SP133_Cov_51
CATAGAGGGTCGCCGGCTCTGGATCACGCTCCCCTAGTCGT => spacer 2
>G1SP145_Cov_60
TTTTTGCCTCATGCTTGGGCGACAGCTTTTGACCAA => spacer 3
>G1SP995_Cov_51
ATGGGATATCTGCTGCCCGCCCGGGGAGATGCTGTCCGAG => spacer 22
>G1SP2337_Cov_60
TCTTGACGATGCGGTTGCCCCGCGCCCTTTTCCAGCC => spacer 40
>G1SP193_Cov_54
AACACCTCAGTAGCACGTCATACGCCGACCAATCATCAG => spacer 12
>G1SP611_Cov_54
ACGACGTTAGGGCATGCAGCATGCCGTCCCCGTTTTTGA => spacer 16
>G1SP617_Cov_57
TGCTCTTGAGCAACGCCATCATCCGGCGCCGCAGCTCCGC => spacer 17
>G1SP1567_Cov_60
TTGGAGCGTGTCACCGCAGACGGCACGATTGAGACAA => spacer 32
>G1SP225_Cov_57
CTGAGGAGAGCGAGTACTCGGGGCTGCCGTCTGCGCTG => spacer 15
>G1SP1545_Cov_57
ACATCCCACGCGTTACCGCTGGCGCGCATCATTCATCGA => spacer 8
>G1SP249_Cov_60
CGGGCAGCGTTCGACACCCGCTCTAGTTGACTTCCGG => spacer 20
>G1SP2436_Cov_48
CAGGTGAGCAACGGCGGCGGCAACCTGGCGGCCACGGGTCG => spacer 21
>G1SP1978_Cov_51
GCATATCGCCCGCCACACCACAGCCACGCTACTGCTCCAT => spacer 66
>G1SP267_Cov_57
TTCGTCGACCATCATTGCCATTCCCTCTCCCCACGT => spacer 23
>G1SP2593_Cov_24
ACACCGCCGATGACAGCTATGTCCGAGTGACATCCTCCCA => spacer 67
>G1SP282_Cov_51
TTGCGCCAACCCTTTCGGTGTGATGCGGATGGTCGGCTCGG => spacer 24
>G1SP300_Cov_54
CCTCAGCTCAGCATCGCTGATGCGGTCCAGCTCGTCCGT => spacer 33
>G1SP315_Cov_12
CCAACCTCACCGCCTGCTGGGTGAGACGTGCTCGCCGCGA => spacer 34
>G1SP3428_Cov_33
TCCACATCGATTTCCTTGACCTCGCCAGGAGAGAAGATCAC => spacer 30
>G1SP1174_Cov_54
TCGTCGACGATCGCGTCGATGTCGATGTCCCAATCGTCGA => spacer 31
>G1SP1221_Cov_57
TTTATCACTCCCGACCAAATAGGTATCGGCGTGTTCAA => spacer 42
>G1SP1581_Cov_57
TCGACACCGACATGACGGCGGTGCCGCACTTGACGCA => spacer 43
>G1SP1824_Cov_39
CTTGAATAACGCGCAGTGAATTTCG => spacer 25

>G1SP904_Cov_72
TCGGGGAGCCGATCAGCGACCACCGCACCCTGTCA => spacer 35
>G1SP2098_Cov_57
CGCATGGACCCGGGCGAGCTGCAGATGGTCCGGGAG => spacer 28
>G1SP2110_Cov_60
TGGATTGCGCTAACTGGCTTGGCGCTGATCCTGGTG => spacer 29
>G1SP402_Cov_54
CTGCATCCGGAAAGTCCGTACGCTCGAAACGCTTCCAACGT => spacer 46

Interpretation – part 2:
+ Mutants of spacer 7 and 52 are found in an adequate manner.
- Missed 2 spacers : 55 and 56. Only first 2/3 of spacer 25 sequence i.e. 25 bp instead of 37bp. Note that these spacers have an IS6110 insertion in their neighborhood.

3. Flanking regions

Command :
crisprtools extract -f crass.crispr

Result :
>G1FL2241
AATGCTGGCGACGATTTTCGCTGTTGTGGTTCTCATTTGAACCGCCCCGGCATGTCCGGA
GACTCCAGTTCTTGGAAAGGATGGGGTCATGTCAG
>G1FL2225
TGAACCGCCCCGGCATGTCCGGAGACTCCAGTTCTTGGAAAGGATGGGGTCATGTCAGG
TGGTTCATCGAGGAGGTACCCGCCGGAGCTGCGTGA

Interpretation – part 3:
Identification of IS6110 near spacer 55.

4. Spacer organization

see scheme below

Interpretation -part 4 :
The locus reconstruction contains 3 contigs, one with a bifurcation and a loop, and the other one with a bifurcation → the structure of the locus cannot be fully resolved.

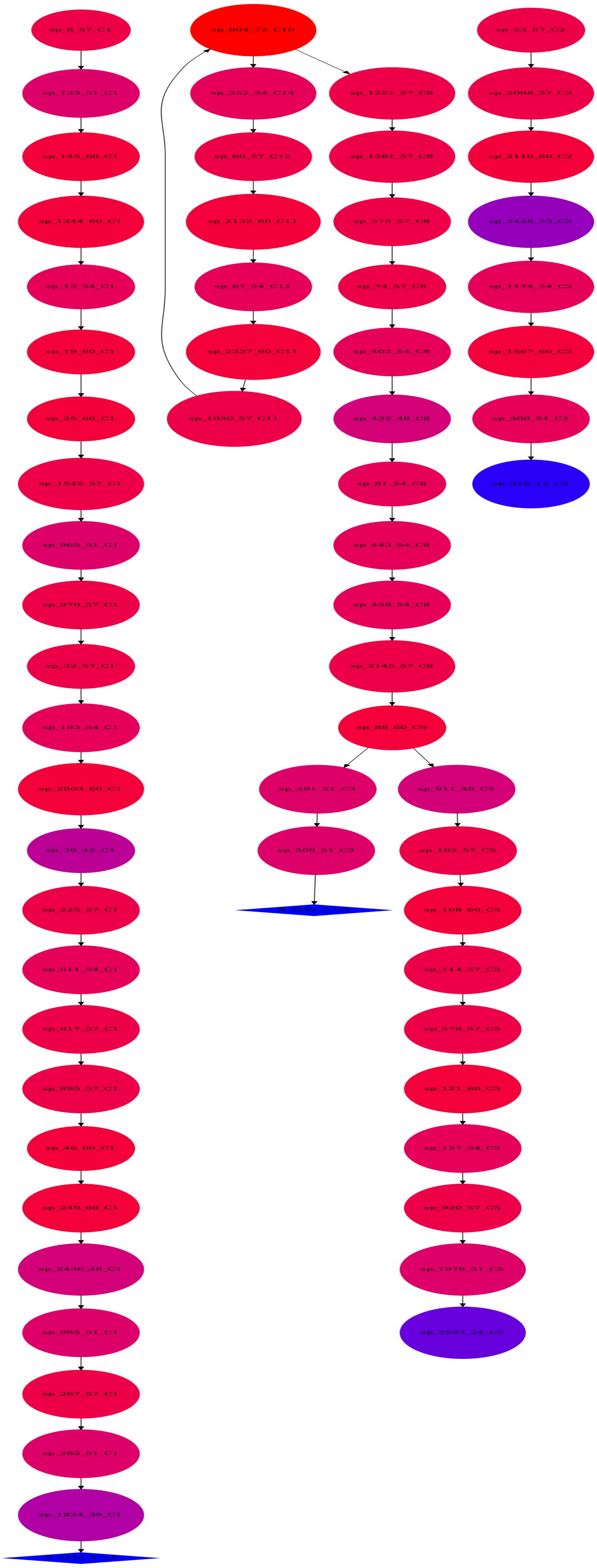
sp_6_57_C1
sp_133_51_C1
sp_145_60_C1
sp_1244_60_C1
sp_13_54_C1
sp_19_60_C1
sp_25_60_C1
sp_1545_57_C1
sp_965_51_C1
sp_970_57_C1
sp_32_57_C1
sp_193_54_C1
sp_2003_60_C1
sp_39_42_C1
sp_225_57_C1
sp_611_54_C1
sp_617_57_C1
sp_895_57_C1
sp_46_60_C1
sp_249_60_C1
sp_2436_48_C1
sp_995_51_C1
sp_267_57_C1
sp_282_51_C1
sp_1824_39_C1
fl_2225_3_C1
sp_904_72_C10
sp_352_54_C14
sp_60_57_C12
sp_2132_60_C11
sp_67_54_C11
sp_2337_60_C11
sp_1030_57_C11
sp_1221_57_C8
sp_1581_57_C8
sp_375_57_C8
sp_74_57_C8
sp_402_54_C8
sp_422_48_C8
sp_81_54_C8
sp_443_54_C8
sp_458_54_C8
sp_2145_57_C8
sp_88_60_C9
sp_491_51_C3
sp_509_51_C3
fl_2241_3_C3
sp_911_48_C5
sp_102_57_C5
sp_108_60_C5
sp_114_57_C5
sp_578_57_C5
sp_121_60_C5
sp_127_54_C5
sp_920_57_C5
sp_1978_51_C5
sp_2593_24_C5
sp_53_57_C2
sp_2098_57_C2
sp_2110_60_C2
sp_3428_33_C2
sp_1174_54_C2
sp_1567_60_C2
sp_300_54_C2
sp_315_12_C2

5. Global interpretation

Only typical DR was identified (misses DR variants), most spacers with their correct variations were identified but two neaby IS insertions were missed. Last, the structure of the locus could not be fully resolved due to IS insertions and duplications.

B.III. CRISPR_detector

Command :
crispr Finder SRA_125_shuffled.fasta 23 45

Results :
Number of frequent kmers is 10960 5480
Number of relevant reads is 1636
STATS
Reads: 0.000331163406372
Stats: 0.318933010101
Refined Stats: 0.0265839099884
Refined Reads: 0.0198240280151
Update Kmers: 0.102835893631
Duplication: 0.0188961029053
Hash Computation: 1.57688784599
Match Kmers: 0.0401830673218
Good Analysis : 0
Bad Analysis: 1.67359018326
Clustering: 1.19209289551e-05
Canonization: 0
Alignment: 0
Total 3.84799385071

Discussion :
No crispr found.

Interpretation :
No ability to detect the CRISPR.

B.IV. CRISPRbuilder-TB

Produced (automatic) results :

('*starting_pattern1*DR0*esp1*DR0*esp2*DR0*esp3*DR0*esp4*DR0*esp5*DR0*esp6*DR0*ATTTCGACGACAATTCGTTGACCACGGATTTTCAGA*DR0*esp8*DR0*esp9*DR0*esp10*DR0*esp11*DR0*esp12*DR0*esp13*GTCGTCAGACCCAAAACCCCGAGAGGGGACGGCAAC*esp14*DR0*esp15*DR0*esp16*DR0*esp17*DR0*esp18*DR0*esp19*DR0*esp20*DR0*esp21*DR0*esp22*DR0*esp23*DR0*esp24*DR0*esp25*AAAACCCCGAGAGGGGACGGAAACTGAACCGCCCCGGCATGTCCGG', 34050)
('CATCGCCGCCTCTACCAGTACTGCGGCGACGTCCCGCCGGTCGAACTCGAGGCTGCCTACTACGCTCAACGCCAGAGACCAGCCGCCGGCTGAGGTCTC*finIS6110*DR0*esp27*DR0*esp28*DR0*esp29*DR0*esp30*DR2*esp31*DR0*esp32*DR0*esp33*DR0*esp34*rDRa1*debIS6110*GGTCATGTCAGGTGGTTCATCGAGGAGGTACCCGCCGGAGCTGCGTGAGCGGGCGGTGCGGATGGTCGCAGAGATCCGCGGTCAGCACGATTCGGAGT', 15336)
('CCGGTCGAACTCGAGGCTGCCTACTACGCTCAACGCCAGAGACCAGCCGCCGGCTGAGGTCTC*finIS6110*DRb1*esp35*DR0*esp36*DR0*esp37*DR0*esp38*DR0*esp39*DR0*esp40*DR0*esp41*DR0*esp35*DR0*esp42*DR0*esp43*DR0*esp44*DR0*esp45*DR0*esp46*DR0*esp47*DR0*esp48*DR0*esp49*DR0*esp50*DR25*esp51*DR0*GCCCCGTGGATGGCGGATGCGTTTTGCGCGCAAGT*DR0*esp53*DR0*esp54*DR0*esp55*debIS6110[:21]', 30273)
('CGGTCGAACTCGAGGCTGCCTACTACGCTCAACGCCAGAGACCAGCCGCCGGCTGAGGTCTC*finIS6110*esp56*DR0*esp57*DR0*esp58*DR0*esp59*DR0*esp60*DR0*esp61*DR0*esp62*DR0*esp63*DR0*esp64*DR0*esp65*DR0*esp66*DR4*esp67*DR5*esp68*DR0*ending_pattern1*Rv2813c[:27]', 19191)

('GTGCTGACCGCGGATCTCTGCGACCATCCGCACCGCCCGCTCACGCAGCTCCGGCGGGTACCTCCTCGATGAACCACCTGACATGACCCCATCCTTTCC*finIS6110c*GTTTCCGTCCCCTCTCGGGGTTTTGGGTCTGATCCGCGAAATTCACTGCGCGTTATTCAAGGTTTCCGTCCCCTCTCGGGGTTTTGGGTCTGACGAC', 2904)
('CGCTCACGCAGCTCCGGCGGGTACCTCCTCGATGAACCACCTGACATGACCCCATCCTTTCC*finIS6110c*GGGTTTTG', 30)
('CCGCTCACGCAGCTCCGGCGGGTACCTCCTCGATGAACCACCTGACATGACCCCATCCTTTCC*finIS6110c*AATGAGAACCACAACAGCGAAAATCGTCGCCAGCATTGTTTCCGTCCCCTCTCGGGGTTTTGGGTCTGACGACATCAACGCAACTCTCATGATCGATG', 1641)

('GGTGAGTCCGGAGACTCTCTGATCTGAGACCTCAGCCGGCGGCTGGTCTCTGGCGTTGAGCGTAGTAGGCAGCCT
CGAGTTCGACCGGCGGGACGTCGCCGCAGTACTGGTAGAGGCGGCGATGGTTGAACCA', 600)

Manual investigation :

Using the 4 first contigs, split at IS6110 positions, we can reconstruct the following CRISPR :

*motif_debut1*DR0*esp1*DR0*esp2*DR0*esp3*DR0*esp4*DR0*esp5*DR0*esp6*DR0*
ATTTCGACGACAATTCGTTGACCACGGATTTTCAGA*DR0*esp8*DR0*esp9*DR0*esp10*DR0*esp11*DR0*
esp12*DR0*esp13*GTCGTCAGACCCAAAACCCCGAGAGGGGACGGCAAC*esp14*DR0*esp15*DR0*esp16*DR0*esp1
7*DR0*esp18*DR0*esp19*DR0*esp20*DR0*esp21*DR0*esp22*DR0*esp23*DR0*esp24*DR0*
esp25*DRb2*IS6110*DR0*esp27*DR0*esp28*DR0*esp29*DR0*esp30*DR2*esp31*DR0*esp32*DR0*
esp33*DR0*esp34*rDRa1*IS6110*DRb1*esp35*DR0*esp36*DR0*esp37*DR0*esp38*DR0*esp39*DR0*
esp40*DR0*esp41*DR0*esp35*DR0*esp42*DR0*esp43*DR0*esp44*DR0*esp45*DR0*esp46*DR0*
esp47*DR0*esp48*DR0*esp49*DR0*esp50*DR25*esp51*DR0*GCCCCGTGGATGGCGGATGCGTTTTGCGCGCAAGT*
DR0*esp53*DR0*esp54*DR0*esp55*IS6110*esp56*DR0*esp57*DR0*esp58*DR0*esp59*DR0*esp60*DR0*esp61*DR0*
esp62*DR0*esp63*DR0*esp64*DR0*esp65*DR0*esp66*DR4*esp67*DR5*esp68*DR0*motif_fin1*Rv2813c

Interpretation :
Full success after simple manual reconstruction. The good order of spacers has been recovered, and the DR variants and IS6110 insertions are exactly the same as in the simulated DNA sequence. New variants for spacers 7 and 52 have been found, as well as new DR variant between spaceurs 13 and 14.

C- Simulation and reconstruction of CRISPR, CRISPR-6

C.I. Simulated data

Evolution characteristics :
0.5% mutation.
Tandem duplications allowed.
IS6110 insertions allowed.

Length reads : 300.

Nucleotidic sequence :
CATCATCAGCAGGCATTGTTACCACACGCTGGACGAATTGTCCATAGAGTCGTCAGACCCAAAACCCCGAGAGGGGACGGAAACTTA
AAACCGTGTTGCACTGCAACCCGGAATTCTTGCACGTCGTCAGACCCAAAACCCCGAGAGGGGACGGAAACCATAGAGGGTCGCC
GGCTCTGGATCACGCTCCCCTAGTCGTGTCGTCAGACCCAAAACCCCGAGAGGGGACGGAAACTTTTTGCCTCATGCTTGGGCGA
CAGCTTTTGACCAAGTCGTCAGACCCAAAACCCCGAGAGGGGACGGAAACTCGCAAGCGCCGTGCTTCCAGTGATCGCCTTCTAGT
CGTCAGACCCAAAACCCCGAGAGGGGACGGAAACTCGCGGCGCGGCATGGCACGGCAGGCGTGGCTAGGGGGTCGTCAGACCC
AAAACCCCGAGAGGGGACGGAAACATGTGCGCCGTCGCCGTAAGTGCCCCACGGCCCGTGTCGTCAGACCCAAAACCCCGAGAG
GGGACGGAAACATTTCGACGACAATTCGTTGACCACGAATTTTCAGAGTCGTCAGACCCAAAACCCCGAGAGGGGACGGAAACACA
TCCCACGCGTTACCGCTGGCGCGCATCATTCATCGAGTCGTCAGACCCAAAACCCCGAGAGGGGACGGAAACCCATATCGGGGAC
GGCGACGCTGCGAGAGGACACGCCGAGTCGTCAGACCCAAAACCCCGAGAGGGGACGGAAACTACACCACGCGTCGTGCCATCA
GTCAGCGTCCTCCTCGTCGTCAGACCCAAAACCCCGAGAGGGGACGGAAACTTGAACACGGAGCCGTGCACATGCCGTGGCTCA
GGGGTGTCGTCAGACCCAAAACCCCGAGAGGGGACGGAAACAACACCTCAGTAGCACGTCATACGCCGACCAATCATCAGGTCGT
CAGACCCAAAACCCCGAGAGGGGACGGAAACTTTTCTGACCACTTGTGCGGGAATAGCGGGCTTAGGTCGTCAGACCCAAAACCC
CGAGAGGGGACGGAAACACCAATGCGTCGTCATTTCCGGCTTCAATTTCAGCCTGTCGTCAGACCCAAAACCCCGAGAGGGGACG
GAAACCTGAGGAGAGCGAGTACTCGGGGCTGCCGTCTGCGCTGGTCGTCAGACCCAAAACCCCGAGAGGGGACGGAAACACGAC
GTTAGGGCATGCAGCATGCCGTCCCCGTTTTTGAGTCGTCAGACCCAAAACCCCGAGAGGGGACGGAAACTGCTCTTGAGCAACG
CCATCATCCGGCGCCGCAGCTCCGCGTCGTCAGACCCAAAACCCCGAGAGGGGACGGAAACGCGTGAAACCGCCCCCAGCCTCG
CCGGGGCCGCCTAGGTCGTCAGACCCAAAACCCCGAGAGGGGACGGAAACACTCGGAATCCCATGTGCTGACAGCGGATTCGCAT
GTCGTCAGACCCAAAACCCCGAGAGGGGACGGAAACCGGGCAGCGTTCGACACCCGCTCTAGTTGACTTCCGGGTCGTCAGACC
CAAAACCCCGAGAGGGGACGGAAACCAGGTGAGCAACGGCGGCGGCAACCTGGCGGCCACGGGTCGGTCGTCAGACCCAAAAC
CCCGAGAGGGGACGGAAACATGGGATATCTGCTGCCCGCCCGGGGAGATGCTGTCCGAGGTCGTCAGACCCAAAACCCCGAGAG
GGGACGGAAACTTCGTCGACCATCATTGCCATTCCCTCTCCCCACGTGTCGTCAGACCCAAAACCCCGAGAGGGGACGGAAACTTG
CGCCAACCCTTTCGGTGTGATGCGGATGGTCGGCTCGGGTCGTCAGACCCAAAACCCCGAGAGGGGACGGAAACCTTGAATAACG
CGCAGTGAATTTCGCGGATCAGACCCAAAACCCCGAGAGGGGACGGAAACATTCGCACGAGTTCCCGCCAGCGTCGTAAATCGCC
AGTCGTCAGACCCAAAACCCCGAGAGGGGACGGAAACCCGGCAACAATCGCGCCGGCCCGCGCGGATGACTCCGGTCGTCAGAC
CCAAAACCCCGAGAGGGGACGGAAACCGCATGGACCCGGGCGAGCTGCAGATGGTCCGGGAGGTCGTCAGACCCAAAACCCCGA
GAGGGGACGGAAACTGGATTGCGCTAACTGGCTTGGCGCTGATCCTGGTGGTCGTCAGACCCAAAACCCCGAGAGGGGACGGAA
ACTCCAAATCGATTTCCTTGACCTCGCCAGGAGAGAAGATCACGTCGTCAGACCCAAAACCCCGAGAGAGGACGGAAACTCGTCGA
CGATCGCGTCGATGTCGATGTCCCAATCGTCGAGTCGTCAGACCCAAAACCCCGAGAGGGGACGGAAACTTGGAGCGTGTCACCG
CAGACGGCACGATTGAGACAAGTCGTCAGACCCAAAACCCCGAGAGGGGACGGAAACCCTCAGCTCAGCATCGCTGATGCGGTCC
AGCTCGTCCGTGTCGTCAGACCCAAAACCCCGAGAGGGGACGGAAACCCAACCTCACCGCCTGCTGGGTGAGACGTGCTCGCCG
CGAGTCGTCAGACCCAAAACCCTGAACCGCCCCGGCATGTCCGGAGACTCCAGTTCTTGGAAAGGATGGGGTCATGTCAGGTGGT
TCATCGAGGAGGTACCCGCCGGAGCTGCGTGAGCGGGCGGTGCGGATGGTCGCAGAGATCCGCGGTCAGCACGATTCGGAGTGG
GCAGCGATCAGTGAGGTCGCCCGTCTACTTGGTGTTGGCTGCGCGGAGACGGTGCGTAAGTGGGTGCGCCAGGCGCAGGTCGAT
GCCGGCGCACGGCCCGGGACCACGACCGAAGAATCCGCTGAGCTGAAGCGCTTAGCGGCGGGACAACGCCGAATTGCGAAGGG
CGAACGCGATTTTAAAGACCGCGTCGGCTTTCTTCGCGGCCGAGCTCGACCGGCCAGCACGCTAATTAACGGTTCATCGCCGATCA
TCAGGGCCACCGCGAGGGCCCCGATGGTTTGCGGTGGGGTGTCGAGTCGATCTGCACACAGCTGACCGAGCTGGGTGTGCCGAT
CGCCCCATCGACCTACTACGACCACATCAACCGGGAGCCCAGCCGCCGCGAGCTGCGCGATGGCGAACTCAAGGAGCACATCAGC
CGCGTCCACGCCGCCAACTACGGTGTTTACGGTGCCCGCAAAGTGTGGCTAACCCTGAACCGTGAGGGCATCGAGGTGGCCAGAT
GCACCGTCGAACGGCTGATGACCAAACTCGGCCTGTCCGGGACCACCCGCGGCAAAGCCCGCAGGACCACGATCGCTGATCCGG
CCACAGCCCGTCCCGCCGATCTCGTCCAGCGCCGCTTCGGACCACCAGCACCTAACCGGCTGTGGGTAGCAGACCTCACCTATGT
GTCGACCTGGGCAGGGTTCGCCTACGTGGCCTTTGTCACCGACGCCTACGTCGCAGGATCCTGGGCTGGCGGGTCGCTTCCACGA
TGGCCACCTCCATGGTCCTCGACGCGATCGAGCAAGCCATCTGGACCCGCCAACAAGAAGGCGTACTCGACCTGAAAGACGTTATC
CACCATACGGATAGGGGATCTCAGTACACATCGATCCGGTTCAGCGAGCGGCTCGCCGAGGCAGGCATCCAACCGTCGGTCGGAG
CGGTCGGAAGCTCCTATGACAATGCACTAGCCGAGACGATCAACGGCCTATACAAGACCGAGCTGATCAAACCCGGCAAGCCCTGG
CGGTCCATCGAGGATGTCGAGTTGGCCACCGCGCGCTGGGTCGACTGGTTCAACCATCGCCGCCTCTACCAGTACTGCGGCGACG
TCCCGCCGGTCGAACTCGAGGCTGCCTACTACGCTCAACGCCAGAGACCAGCCGCCGGCTGAGGTCTCAGATCAGAGAGTCTCCG
GACTCACCGGGGCGGTTCACCCCGAGAGGGGACGGAAACTCGGGGAGCCGATCAGCGACCACCGCACCCTGTCAGTCGTCAGAC
CCAAAACCCCGAGAGGGGACGGAAACCTTCAGCACCACCATCATCCGGCGCCTCAGCTCAGCATGTCGTCAGACCCAAAACCCCG
AGAGGGGACGGAAACCCTTCGACGCCGGATTCGTGATCTCTTCCCGCGGATAGGTCGTCAGACCCAAAACCCCGAGAGGGGACGG
AAACCCTTCGACGCCGGATTCGTGATCTCTTCCCGCGGATAGGTCGTCAGACCCAAAACCCCGAGAGGGGACGGAAACTGCCCCG
GCGTTTAGCGATCACAACACCAACTAATGGTCGTCAGACCCAAAACCCCGAGAGGGGACGGAAACCAGCGAAATACAGGCTCCACG
ACACGACCACAACGCGTCGTCAGACCCAAAACCCCGAGAGGGGACGGAAACTCTTGACGATGCGGTTGCCCCGCGCCCTTTTCCA
GCCGTCGTCAGACCCAAAACCCCGAGAGGGGACGGAAACAGGTTCGCGTCAGACAGGTTCGCGTCGATCAAGTCCGGTCGTCAG
ACCCAAAACCCCGAGAGGGGACGGAAACTCGGGGAGCCGATCAGCGACCACCGCACCCTGTCAGTCGTCAGACCCAAAACCCCG
AGAGGGGACGGAAACTTTATCACTCCCGACCAAATAGGTATCGGCGTGTTCAAGTCGTCAGACCCAAAACCCCGAGAGGGGACGGA
AACTCGACACCGACATGACGGCGGTGCCGCACTTGACGCAGTCGTCAGACCCAAAACCCCGAGAGGGGACGGAAACCTTTGCGAA
GTCACCTCGCCCACACCGTCGAAGCGCCTGTCGTCAGACCCAAAACCCCGAGAGGGGACGGAAACGCGGATGGTGGGCAAGTTG
GCGCTGGGGTCTGAGTCAAGTCGTCAGACCCAAAACCCCGAGAGGGGACGGAAACCTGCATCCGGAAAGTCCGTACGCTCGAAAC
GCTTCCAACGTGTCGTCAGACCCAAAACCCCGAGAGGGGACGGAAACTCGAAATCCAGCACCACATCCGCAGCTGCGGCATGCTC
CCGAAGTCGTCAGACCCAAAACCCCGAGAGGGGACGGAAACGCGAGGAACCGTCCCACCTGGGCCTGCCCCCAGCGGGTCGTCA
GACCCAAAACCCCGAGAGGGGACGGAAACTCAATAACACTTTTTTTGAGCGTGGCGCGGTTGAGAGTGTCGTCAGACCCAAAACCC

CGAGAGGGGACGGAAACACGGAAACGCAGCACCAGCCTGACAATCTTATTCTCGCGTCGTCAGACCCAAAACCCCGAGAGGGGAC
GGAAACATTTTGAGCGCGAACTCGTCCACAGTCCCCCTTTCAGGTCGTCAGACCCAAAACCCCGAGAGGGGACGGAAACGCCCCG
TGGATGGCGGATGCGTTGTGCGCGCAAGTGTCGTCAGACCCAAAACCCCGAGAGGGGACGGAAACCCGACGATGGCCAGTAAATC
GGCGTGGGTAACCGATCCGGGTCGTCAGACCCAAAACCCCGAGAGGGGACGGAAACCCTCGCAGAAAAGGGCATCGATCATGAGA
GTTGCGTTGATGTCGTCAGACCCAAAACCCCGCGAGGGGACGGAAACAATGCTGGCGACGATTTTCGCTGTTGTGGTTCTCATTGT
CGTCAGACCCAAAACCCCGAGAGGGGACGGAAACGCACACCAGCACCTCCCTTGACAATCCGGCAGATCAGACGTCGTCAGACCC
AAAACCCCGAGAGGGGACGGAAACTCGCGGGCTCTGGCCTAAGGGTGCTGACTTCGCCTGTAGTCGTCAGACCCAAAACCCCGAG
AGGGGACGGAAACCCGACGACGAGCAGCGGCATACAGAGCCACGGATACGCCAGGTCGTCAGACCCAAAACCCCGAGAGGGGAC
GGAAACTTGCATCCACTCGTCGCCGACACGGCGGACTTCCGCGAGTCGTCAGACCCAAAACCCCGAGAGGGGACGGAAACTGGTA
ATTGCGTCACGGCTCGCCTGGCGGGCCGATTGTCGTCAGACCCAAAACCCCGAGAGGGGACGGAAACACCATCCGACGCAGGCA
CCGAAGTCGATGACAAGCCGTCGTCAGACCCAAAACCCCGAGAGGGGACGGAAACTAGTACGCCATCTGTGCCTCATACAGGTCCA
GTGCCCTGTCGTCAGACCCAAAACCCCGAGAGGGGACGGAAACCTGACGGCACGGAGCTTTCCGGCTTCTATCAGGTAGTCGTCA
GACCCAAAACCCCGAGAGGGGACGGAAACCCTCATGGTGGGACATGGACGAGCGCGACTATCGGGGTCGTCAGACCCAAAACCC
CGAGAGGGGACGGAAACTGGACGCAGAATCGCACCGGGTGCGGGAGGTGCAGCAGTCGTCAGACCCAAAACCCCGAGAGGGGA
CGGAAACGCATATCGCCCGCCACACCACAGCCACGCTACTGCTCCATGTCCTCAGACCCAAAACCCCGAGAGGGGACGGAAACAC
ACCGCCGATGACAGCTATGTCCGAGTGACATCCTCCCAGTCGTCAGACCCAAAACCACGAGAGGGGACGGAAACTTGAACCGCCC
TTCGCGCGGTGTTTCGGCCGTGCCCGAGTCGTCAGACCCAAAACCCCGAGAGGGGACGGAAACTACGACGACTGGGTCGCCACC
GCGTCTGTTGACCGGCATTCAGGATGATCAGTCTGCCGTGACTTCGGCGATGGCGG

CRISPR annotation : ['motif_debut1', 'DR0', 'esp1', 'DR0', 'esp2', 'DR0', 'esp3', 'DR0', 'esp4', 'DR0', 'esp5', 'DR0', 'esp6', 'DR0', 'esp7', 'DR0', 'esp8', 'DR0', 'esp9', 'DR0', 'esp10', 'DR0', 'esp11', 'DR0', 'esp12', 'DR0', 'esp13(var)', 'DR0', 'esp14', 'DR0', 'esp15', 'DR0', 'esp16', 'DR0', 'esp17', 'DR0', 'esp18', 'DR0', 'esp19', 'DR0', 'esp20', 'DR0', 'esp21', 'DR0', 'esp22', 'DR0', 'esp23', 'DR0', 'esp24', 'DR0', 'esp25', 'DRb2', 'esp26(var)', 'DR0', 'esp27', 'DR0', 'esp28', 'DR0', 'esp29', 'DR0', 'esp30(var)', 'DR2', 'esp31', 'DR0', 'esp32', 'DR0', 'esp33', 'DR0', 'esp34', 'rDRa1', 'IS6110', 'DRb1', 'esp35', 'DR0', 'esp36', 'DR0', 'esp37', 'DR0', 'esp37', 'DR0', 'esp38', 'DR0', 'esp39', 'DR0', 'esp40', 'DR0', 'esp41', 'DR0', 'esp35', 'DR0', 'esp42', 'DR0', 'esp43', 'DR0', 'esp44', 'DR0', 'esp45', 'DR0', 'esp46', 'DR0', 'esp47', 'DR0', 'esp48', 'DR0', 'esp49', 'DR0', 'esp50', 'DR0', 'esp51', 'DR0', 'esp52', 'DR0', 'esp53', 'DR0', 'esp54', 'DRv', 'esp55', 'DR0', 'esp56', 'DR0', 'esp57', 'DR0', 'esp58', 'DR0', 'esp59', 'DR0', 'esp60(var)', 'DR0', 'esp61', 'DR0', 'esp62', 'DR0', 'esp63', 'DR0', 'esp64', 'DR0', 'esp65', 'DR0', 'esp66', 'DR4', 'esp67', 'DR5', 'esp68', 'DR0', 'motif_fin1', 'Rv2813c']

Comments :

=> no tandem duplications, 4 spacer variants, 2 DR variants.

C.II. CRASS results

1. CRISPR detection

Command :
./crass SRA_300_shuffled.fasta

Result :
[crass_patternFinder]: Processed 4776 ...0 sec
[crass_clusterCore]: 45 variants mapped to 1 clusters
[crass_clusterCore]: creating non-redundant set
[crass_clusterCore]: 16 non-redundant patterns.
[crass_singletonFinder]: Processed 4776 ...0 sec
[crass_patternFinder]: Found 3858 reads
[crass_graphBuilder]: 1 CRISPRs found!

2. DR investigation

Command :
crisprtools extract -d crass.crispr

Result :
>G1DR1
GTCGTCAGACCCAAAACCCCGAGAGGGGACGGAAAC

Interpretation – part 1:
DR0 has been recovered well, but no variant found.

3. Spacer investigation

Command :
crisprtools extract -s crass.crispr

Result :
>G1SP941_Cov_180
TCGCGGCGCGGCATGGCACGGCAGGCGTGGCTAGGGG => spacer 5
>G1SP2827_Cov_69
ACACCGCCGATGACAGCTATGTCCGAGTGACATCCTCCCA => spacer 67
>G1SP6_Cov_60
TTAAAACCGTGTTGCACTGCAACCCGGAATTCTTGCAC => spacer 1
>G1SP12_Cov_171
TTTTTGCCTCATGCTTGGGCGACAGCTTTTGACCAA => spacer 3
>G1SP835_Cov_189
TCGCAAGCGCCGTGCTTCCAGTGATCGCCTTCTA => spacer 4
>G1SP889_Cov_186
AACACCTCAGTAGCACGTCATACGCCGACCAATCATCAG => spacer 12
>G1SP970_Cov_189
ATTTCGACGACAATTCGTTGACCACGAATTTTCAGA => spacer 7
>G1SP35_Cov_183
ACATCCCACGCGTTACCGCTGGCGCGCATCATTCATCGA => spacer 8
>G1SP38_Cov_177
CCATATCGGGGACGGCGACGCTGCGAGAGGACACGCCGA => spacer 9
>G1SP41_Cov_174
TACACCACGCGTCGTGCCATCAGTCAGCGTCCTCCTC => spacer 10
>G1SP886_Cov_189
TTGAACACGGAGCCGTGCACATGCCGTGGCTCAGGGGT => spacer 11
>G1SP57_Cov_192
TTTTCTGACCACTTGTGCGGGAATAGCGGGCTTAG => spacer 13
>G1SP60_Cov_189
ACCAATGCGTCGTCATTTCCGGCTTCAATTTCAGCCT => spacer 14
>G1SP63_Cov_186
CTGAGGAGAGCGAGTACTCGGGGCTGCCGTCTGCGCTG => spacer 15
>G1SP66_Cov_186
ACGACGTTAGGGCATGCAGCATGCCGTCCCCGTTTTTGA => spacer 16
>G1SP944_Cov_189
ATGTGCGCCGTCGCCGTAAGTGCCCCACGGCCCGT => spacer 6
>G1SP1401_Cov_186
GCGAGGAACCGTCCCACCTGGGCCTGCCCCCAGCGG => spacer 48
>G1SP1128_Cov_189
TGCTCTTGAGCAACGCCATCATCCGGCGCCGCAGCTCCGC => spacer 17
>G1SP541_Cov_189
ATTTTGAGCGCGAACTCGTCCACAGTCCCCCTTTCAG => spacer 51
>G1SP544_Cov_192
GCCCCGTGGATGGCGGATGCGTTGTGCGCGCAAGT => spacer 52
>G1SP547_Cov_159
CCGACGATGGCCAGTAAATCGGCGTGGGTAACCGATCCGG => spacer 53
>G1SP550_Cov_129
CCTCGCAGAAAAGGGCATCGATCATGAGAGTTGCGTTGAT => spacer 54
>G1SP121_Cov_183
GCGTGAAACCGCCCCCAGCCTCGCCGGGGCCGCCTAG => spacer 18
>G1SP124_Cov_192
ACTCGGAATCCCATGTGCTGACAGCGGATTCGCAT => spacer 19
>G1SP127_Cov_189
CGGGCAGCGTTCGACACCCGCTCTAGTTGACTTCCGG => spacer 20
>G1SP1452_Cov_186
TCAATAACACTTTTTTTGAGCGTGGCGCGGTTGAGAGT => spacer 49
>G1SP607_Cov_108
AATGCTGGCGACGATTTTCGCTGTTGTGGTTCTCATT => spacer 55
>G1SP181_Cov_186
ATGGGATATCTGCTGCCCGCCCGGGGAGATGCTGTCCGAG => spacer 22
>G1SP4057_Cov_15
TTGAACCGCCCTTCGCGCGGTGTTTCGGCCGTGCCCGA => spacer 68
>G1SP1458_Cov_189
ACGGAAACGCAGCACCAGCCTGACAATCTTATTCTCGC => spacer 50
>G1SP640_Cov_126
GCACACCAGCACCTCCCTTGACAATCCGGCAGATCAGAC => spacer 56
>G1SP203_Cov_105
ATTCGCACGAGTTCCCGCCAGCGTCGTAAATCGCCA => spacer 26
>G1SP206_Cov_144
CCGGCAACAATCGCGCCGGCCCGCGCGGATGACTCCG => spacer 27
>G1SP209_Cov_186
CGCATGGACCCGGGCGAGCTGCAGATGGTCCGGGAG => spacer 28
>G1SP230_Cov_168

TGGATTGCGCTAACTGGCTTGGCGCTGATCCTGGTG => spacer 29
>G1SP643_Cov_159
TCGCGGGCTCTGGCCTAAGGGTGCTGACTTCGCCTGTA => spacer 57
>G1SP654_Cov_186
TTGCATCCACTCGTCGCCGACACGGCGGACTTCCGCGA => spacer 59
>G1SP241_Cov_135
TCGTCGACGATCGCGTCGATGTCGATGTCCCAATCGTCGA => spacer 31
>G1SP244_Cov_162
TTGGAGCGTGTCACCGCAGACGGCACGATTGAGACAA => spacer 32
>G1SP247_Cov_129
CCTCAGCTCAGCATCGCTGATGCGGTCCAGCTCGTCCGT => spacer 33
>G1SP256_Cov_45
CCAACCTCACCGCCTGCTGGGTGAGACGTGCTCGCCGCGA => spacer 34
>G1SP909_Cov_189
TGGTAATTGCGTCACGGCTCGCCTGGCGGGCCGATT => spacer 60
>G1SP691_Cov_186
ACCATCCGACGCAGGCACCGAAGTCGATGACAAGCC => spacer 61
>G1SP694_Cov_189
TAGTACGCCATCTGTGCCTCATACAGGTCCAGTGCCCT => spacer 62
>G1SP697_Cov_189
CTGACGGCACGGAGCTTTCCGGCTTCTATCAGGTA => spacer 63
>G1SP700_Cov_186
CCTCATGGTGGGACATGGACGAGCGCGACTATCGGG => spacer 64
>G1SP754_Cov_186
TGGACGCAGAATCGCACCGGGTGCGGGAGGTGCAGCA => spacer 65
>G1SP312_Cov_243
TCGGGGAGCCGATCAGCGACCACCGCACCCTGTCA => spacer 35
>G1SP315_Cov_138
CTTCAGCACCACCATCATCCGGCGCCTCAGCTCAGCAT => spacer 36
>G1SP318_Cov_309
CCTTCGACGCCGGATTCGTGATCTCTTCCCGCGGATAG => spacer 37
>G1SP1185_Cov_162
TTCGTCGACCATCATTGCCATTCCCTCTCCCCACGT => spacer 23
>G1SP351_Cov_168
TGCCCCGGCGTTTAGCGATCACAACACCAACTAATG => spacer 38
>G1SP354_Cov_186
CAGCGAAATACAGGCTCCACGACACGACCACAACGC => spacer 39
>G1SP1694_Cov_123
GCATATCGCCCGCCACACCACAGCCACGCTACTGCTCCAT => spacer 66
>G1SP357_Cov_189
TCTTGACGATGCGGTTGCCCCGCGCCCTTTTCCAGCC => spacer 40
>G1SP414_Cov_189
AGGTTCGCGTCAGACAGGTTCGCGTCGATCAAGTCCG => spacer 41
>G1SP420_Cov_189
TTTATCACTCCCGACCAAATAGGTATCGGCGTGTTCAA => spacer 42
>G1SP423_Cov_186
TCGACACCGACATGACGGCGGTGCCGCACTTGACGCA => spacer 43
>G1SP426_Cov_189
CTTTGCGAAGTCACCTCGCCCACACCGTCGAAGCGCCT => spacer 44
>G1SP429_Cov_189
GCGGATGGTGGGCAAGTTGGCGCTGGGGTCTGAGTCAA => spacer 45

Interpretation – part 2:
+ Mutants of spacer 13, 23, and 60 are found in an adequate manner.
- Missed 8 spacers, and no information about spacer duplication.

3. Flanking regions

Command :
crisprtools extract -f crass.crispr

Result :
>G1FL9
CATAGAGGGTCGCCGGCTCTGGATCACGCTCCCCTAGTCGT
>G1FL480
CTGCATCCGGAAAGTCCGTACGCTCGAAACGCTTCCAACGT
>G1FL492
TCGAAATCCAGCACCACATCCGCAGCTGCGGCATGCTCCCGAA

>G1FL645
CCGACGACGAGCAGCGGCATACAGAGCCACGGATACGCCAG
>G1FL130
CAGGTGAGCAACGGCGGCGGCAACCTGGCGGCCACGGGTCG
>G1FL2994
CTTGAATAACGCGCAGTGAATTTCG
>G1FL1194
TTGCGCCAACCCTTTCGGTGTGATGCGGATGGTCGGCTCGG
>G1FL1255
TCCAAATCGATTTCCTTGACCTCGCCAGGAGAGAAGATCAC

Interpretation – part 3:
Some spacers are identified as flanking sequences :
>G1FL9 -> esp2
>G1FL480 -> esp46
>G1FL492 -> esp47
>G1FL645 -> esp58
>G1FL130 -> esp21
>G1FL2994 -> esp25 (begin)
>G1FL1194 -> esp24
>G1FL1255 -> esp30 (mutated)

4. **Spacer organization**

see scheme below

Interpretation – part 4 :

Two contigs with one having seevral mutlifurcations (See figure below) : Crass failed in recovering the overall structure of this locus.

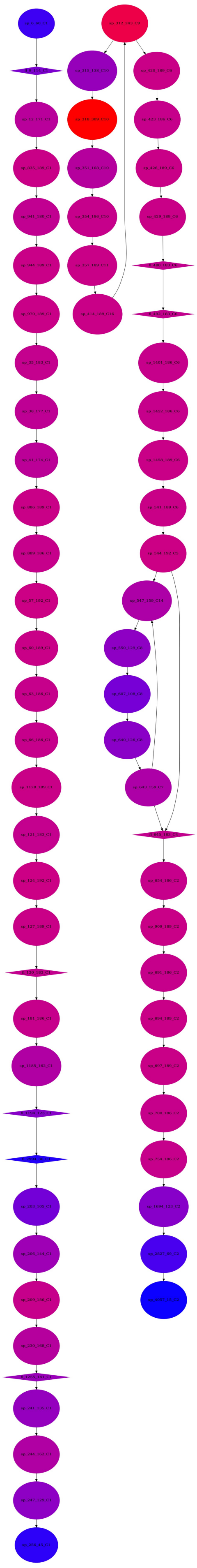

C.III. CRISPR_detector

Command :
crispr Finder SRA_300_shuffled.fasta 23 45

Results :
Number of frequent kmers is 10760 5380
Number of relevant reads is 1610
STATS
Reads: 0.000331163406372
Stats: 2.98384213448
Refined Stats: 0.104677200317
Refined Reads: 0.0799968242645
Update Kmers: 0.913692951202
Duplication: 0.198436021805
Hash Computation: 10.3141438961
Match Kmers: 0.170535802841
Good Analysis : 0
Bad Analysis: 38.4325861931
Clustering: 2.69412994385e-05
Canonization: 0
Alignment: 0
Total 53.7512481213

Discussion :
No crispr found.

Interpretation :
No ability to detect the CRISPR.

C.IV. CRISPRbuilder

Produced (automatic) results :

('*starting_pattern1*DR0*esp1*DR0*esp2*DR0*esp3*DR0*esp4*DR0*esp5*DR0*esp6*DR0*esp7*DR0*esp8*DR0*esp9*
DR0*esp10*DR0*esp11*DR0*esp12*DR0*TTTTCTGACCACTTGTGCGGGAATAGCGGGCTTAG*DR0*esp14*DR0*esp1
5*DR0*esp16*DR0*esp17*DR0*esp18*DR0*esp19*DR0*esp20*DR0*esp21*DR0*esp22*DR0*esp23*DR0*esp24*DR0*e
sp25*AAAACCCCGAGAGGGGACGGAAACATTCGCACGAGTTCCCGCCAGCGTCGTAAATCGCCA*DR0*esp27*DR0*
esp28*DR0*esp29*DR0*TCCAAATCGATTTCCTTGACCTCGCCAGGAGAGAAGATCAC*DR2*esp31*DR0*esp32*DR0*e
sp33*DR0*esp34*rDRa1*deb**IS6110***GGTCATGTCAGGTGGTTCATCGAGGAGGTACCCGCCGGAGCTGCGTGAGCGG
GCGGTGCGGATGGTCGCAGAGATCCGCGGTCAGCACGATTCGGAGTGGGCAGCGATCAGTGAGGTCGCCCGTCTA
CTTGGTGTTGGCTGCGCGGAGACGGTGCGTAAGTGGGTGCGCCAGGCGCAGGTCGATGCCGGCGCACGGCCCGG
GACCACGACCGAAGAATCCGCTGAGCTGAAGCGCTTAGCGGCGGGACAACGCCGAATTGCGAAGGGCG', 115380)

('CTCGCCGAGGCAGGCATCCAACCGTCGGTCGGAGCGGTCGGAAGCTCCTATGACAATGCACTAGCCGAGACGATC
AACGGCCTATACAAGACCGAGCTGATCAAACCCGGCAAGCCCTGGCGGTCCATCGAGGATGTCGAGTTGGCCACCG
CGCGCTGGGTCGACTGGTTCAACCATCGCCGCCTCTACCAGTACTGCGGCGACGTCCCGCCGGTCGAACTCGAGG
CTGCCTACTACGCTCAACGCCAGAGACCAGCCGCCGGCTGAGGTCTC***finIS6110***DRb1*esp35*DR0*esp36*DR0*es
p37*DR0*esp37*DR0*esp38*DR0*esp39*DR0*esp40*DR0*esp41*DR0*esp35*DR0*esp42*DR0*esp43*DR0*esp44*DR
0*esp45*DR0*esp46*DR0*esp47*DR0*esp48*DR0*esp49*DR0*esp50*DR0*esp51*DR0*esp52*DR0*esp53*DR0*esp54
*GTCGTCAGACCCAAAACCCCGCGAGGGGACGGAAAC*esp55*DR0*esp56*DR0*esp57*DR0*esp58*DR0*esp59*DR
0*TGGTAATTGCGTCACGGCTCGCCTGGCGGGCCGATT*DR0*esp61*DR0*esp62*DR0*esp63*DR0*esp64*DR0*esp6
5*DR0*esp66*DR4*esp67*DR5*esp68*DR0***ending_pattern1***Rv2813c[:24]', 124200)

('CGCAATTCGGCGTTGTCCCGCCGCTAAGCGCTTCAGCTCAGCGGATTCTTCGGTCGTGGTCCCGGGCCGTGCGCC
GGCATCGACCTGCGCCTGGCGCACCCACTTACGCACCGTCTCCGCGCAGCCAACACCAAGTAGACGGGCGACCTC
ACTGATCGCTGCCCACTCCGAATCGTGCTGACCGCGGATCTCTGCGACCATCCGCACCGCCCGCTCACGCAGCTCC
GGCGGGTACCTCCTCGATGAACCACCTGACATGACCCCATCCTTTCC*finIS6110c*esp81[19:24]', 900)
('TGAGTCCGGAGACTCTCTGATCTGAGACCTCAGCCGGCGGCTGGTCTCTGGCGTTGAGCGTAGTAGGCAGCCTCG
AGTTCGACCGGCGGGACGTCGCCGCAGTACTGGTAGAGGCGGCGATGGTTGAACCAGTCGACCCAGCGCGCGGT
GGCCAACTCGACATCCTCGATGGACCGCCAGGGCTTGCCGGGTTTGATCAGCTCGGTCTTGTATAGGCCGTTGATC
GTCTCGGCTAGTGCATTGTCATAGGAGCTTCCGACCGCTCCGACCGACGGTTGGATGCCTGCCTCGGCGAGCCGCT
CG', 360)

Manual investigation :

The two first contigs reproduce the whole CRISPR locus divided in two due to the IS6110. Duplication of spacer 37 has been automatically detected, as well as all variants of spacers and Drs.

*starting_pattern1*DR0*esp1*DR0*esp2*DR0*esp3*DR0*esp4*DR0*esp5*DR0*esp6*DR0*esp7*DR0*esp8*DR0*esp9*DR0*esp10*DR0*esp11*DR0*esp12*DR0*esp13(var)*DR0*esp14*DR0*esp15*DR0*esp16*DR0*esp17*DR0*esp18*DR0*esp19*DR0*esp20*DR0*esp21*DR0*esp22*DR0*esp23*DR0*esp24*DR0*esp25*DRv*esp26(var)*DR0*esp27*DR0*esp28*DR0*esp29*DR0*esp30(var)*DR2*esp31*DR0*esp32*DR0*esp33*DR0*esp34*rDRa1*IS6110*DRb1*esp35*DR0*esp36*DR0*esp37*DR0*esp37*DR0*esp38*DR0*esp39*DR0*esp40*DR0*esp41*DR0*esp35*DR0*esp42*DR0*esp43*DR0*esp44*DR0*esp45*DR0*esp46*DR0*esp47*DR0*esp48*DR0*esp49*DR0*esp50*DR0*esp51*DR0*esp52*DR0*esp53*DR0*esp54*DRv*esp55*DR0*esp56*DR0*esp57*DR0*esp58*DR0*esp59*DR0*esp60(var)*DR0*esp61*DR0*esp62*DR0*esp63*DR0*esp64*DR0*esp65*DR0*esp66*DR4*esp67*DR5*esp68*DR0*ending_pattern1*Rv2813c

Interpretation :

Full success after simple manual reconstruction. The good order of spacers has been recovered, and the DR variants and IS6110 insertions are exactly the same as in the simulated DNA sequence. New variants for spacers 7 and 52 have been found, as well as new DR variant between spaceurs 13 and 14.
