# Supplementary material for: CRISPRbuilder-TB: “CRISPR-builder for tuberculosis”. Exhaustive reconstruction of the CRISPR locus in mycobacterium tuberculosis complex using SRA
Source: PLoS Comput Biol. 2021 Mar 5;17(3):e1008500. doi: 10.1371/journal.pcbi.1008500 (PMC7968741; doi:10.1371/journal.pcbi.1008500)
Supplement: S2 Text — (PDF) [file pcbi.1008500.s010.pdf]

S2 Text **Comparative Crass and CRISPR-TB results obtained on two genomes ERR751335 (L5) and SRR6407486 (L4.9)**

A. **Comparison between Crass and CRISPR_builder on ERR751335 genome (L5) CRISPR locus reconstruction**

Crass result

crisprtools extract -s crass.crispr correspondance

>G75SP5_Cov_22 -> esp65
TGGACGCAGAATCGCACCGGGTGCGGGAGGTGCAGCA
>G75SP10_Cov_24 -> 41
AGGTTCGCGTCAGACAGGTTCGCGTCGATCAAGTCCG
>G75SP16_Cov_35 -> 27 muté
CCGGCAACAATCGCGCCGGCCCGAGCGGATGACTCCG
>G75SP20_Cov_30 -> 14
ACCAATGCGTCGTCATTTCCGGCTTCAATTTCAGCCT
>G75SP25_Cov_13 -> 5
TCGCGGCGCGGCATGGCACGGCAGGCGTGGCTAGGGG
>G75SP29_Cov_23 -> 43
TCGACACCGACATGACGGCGGTGCCGCACTTGACGCA
>G75SP33_Cov_30 -> 10
TACACCACGCGTCGTGCCATCAGTCAGCGTCCTCCTC
>G75SP40_Cov_23 -> 40
TCTTGACGATGCGGTTGCCCCGCGCCCTTTTCCAGCC
>G75SP486_Cov_27 -> 23
TTCGTCGACCATCATTGCCATTCCCTCTCCCCACGT
>G75SP50_Cov_25 -> 63
CTGACGGCACGGAGCTTTCCGGCTTCTATCAGGTA
>G75SP54_Cov_24 -> 36
CTTCAGCACCACCATCATCCGGCGCCTCAGCTCAGCAT
>G75SP58_Cov_31 -> 4
TCGCAAGCGCCGTGCTTCCAGTGATCGCCTTCTA
>G75SP63_Cov_29 -> 6
ATGTGCGCCGTCGCCGTAAGTGCCCCACGGCCCGT
>G75SP67_Cov_22 -> 64
CCTCATGGTGGGACATGGACGAGCGCGACTATCGGG
>G75SP71_Cov_13 -> 39
CAGCGAAATACAGGCTCCACGACACGACCACAACGC
>G75SP85_Cov_23 -> 3
TTTTTGCCTCATGCTTGGGCGACAGCTTTTGACCAA
>G75SP79_Cov_26 -> 44
CTTTGCGAAGTCACCTCGCCCACACCGTCGAAGCGCCT
>G75SP529_Cov_12 -> esp2
CATAGAGGGTCGCCGGCTCTGGATCACGCTCCCCTAGTCGT
>G75SP90_Cov_31 -> 7
ATTTCGACGACAATTCGTTGACCACGAATTTTCAGA
>G75SP100_Cov_35 -> 38
TGCCCCGGCGTTTAGCGATCACAACACCAACTAATG

>G75SP104_Cov_26 -> 61
ACCATCCGACGCAGGCACCGAAGTCGATGACAAGCC
>G75SP109_Cov_26 -> 48
GCGAGGAACCGTCCCACCTGGGCCTGCCCCCAGCGG
>G75SP548_Cov_20 -> 17
TGCTCTTGAGCAACGCCATCATCCGGCGCCGCAGCTCCGC
>G75SP129_Cov_31 -> 13
TTTTCTGACCACTTGTGCGGGATTAGCGGGCTTAG
>G75SP134_Cov_24 -> 35
TCGGGGAGCCGATCAGCGACCACCGCACCCTGTCA
>G75SP139_Cov_23 -> 50
ACGGAAACGCAGCACCAGCCTGACAATCTTATTCTCGC
>G75SP144_Cov_25 -> 12
AACACCTCAGTAGCACGTCATACGCCGACCAATCATCAG
>G75SP150_Cov_33 -> 49
TCAATAACACTTTTTTTGAGCGTGGCGCGGTTGAGAGT
>G75SP154_Cov_21 -> 8
ACATCCCACGCGTTACCGCTGGCGCGCATCATTCATCGA
>G75SP159_Cov_26 -> 15
CTGAGGAGAGCGAGTACTCGGGGCTGCCGTCTGCGCTG
>G75SP452_Cov_26 -> 37
CCTTCGACGCCGGATTCGTGATCTCTTCCCGCGGATAG
>G75SP180_Cov_27 -> 26
ATTCGCACGAGTTCCCGTCAGCGTCGTAAATCGCCA
>G75SP187_Cov_30 -> 1
TTAAAACCGTGTTGCACTGCAACCCGGAATTCTTGCAC
>G75SP624_Cov_22 -> 28
CGCATGGACCCGGGCGAGCTGCAGATGGTCCGGGAG
>G75SP197_Cov_21 -> 11
TTGAACACGGAGCCGTGCACATGCCGTGGCTCAGGGGT
>G75SP902_Cov_1 -> 68 mutated
TTGAACAGCCCTTCGCGCGGAGTTTCGGCCGTGCCCGA
TTGAACCGCCCTTCGCGCGGTGTTTCGGCCGTGCCCGA
>G75SP206_Cov_21 -> 67
ACACCGCCGATGACAGCTATGTCCGAGTGACATCCTCCCA
>G75SP216_Cov_1 -> 66 mutated
CCATATCGCCCGCCACACCACAGCCACGCTACTGCTCCAT
>G75SP221_Cov_21 -> 66
GCATATCGCCCGCCACACCACAGCCACGCTACTGCTCCAT
>G75SP640_Cov_33 -> 42(1)
TTTATCACTCCCGACCAAATAGATATCGGCGTGTTCAA
>G75SP990_Cov_18 -> 9
CCATATCGGGGACGGCGACGCTGCGAGAGGACACGCCGA
>G75SP440_Cov_19 -> 62
TAGTACGCCATCTGTGCCTCATACAGGTCCAGTGCCCT
>G75SP871_Cov_18 -> 25 truncated
CTTGAATAACGCGCAGTGAATTTCG
>G75SP273_Cov_21 -> 24
TTGCGCCAACCCTTTCGGTGTGATGCGGATGGTCGGCTCGG
>G75SP284_Cov_32 -> 16

ACGACGTTAGGGCATGCAGCATGCCGTCCCCGTTTTTGA
>G75SP325_Cov_23 -> 45
GCGGATGGTGGGCAAGTTGGCGCTGGGGTCTGAGTCAA
>G75SP329_Cov_28 -> 47
TCGAAATCCAGCACCACATCCGCAGCTGCGGCATGCTCCCGAA
>G75SP349_Cov_29 -> 46
CTGCATCCGGAAAGTCCGTACGCTCGAAACGCTTCCAACGT

ERR751335. (CRISPR-builder)

*Rv2816c*motif_debut1*DR0*esp1*DR0*esp2*DR0*esp3*DR0*esp4*DR0*esp5*DR0*
esp6*DR0*esp7*DR0*esp8*DR0*esp9*DR0*esp10*DR0*esp11*DR0*esp12*DR0*
esp13*DR0*esp14*DR0*esp15*DR0*esp16*DR0*esp17*DR0*esp23*DR0*esp24*DR0*
esp25*DRb2*esp26*DR0*esp27*DR0*esp28*DR0*esp36*DR0*esp37*DR0*
esp38*DR0*esp39*DR0*esp40*DR0*esp41*DR0*esp35*DR0*esp42(1)*DR0*esp43*DR0*
esp44*DR0*esp45*DR0*esp46*DR0*esp47*DR0*esp48*DR0*esp49*DR0*esp50*DR0*
esp61*DR0*esp62*DR0*esp63*DR0*esp64*DR0*esp65*DR0*esp66*DR4*esp67*DR5*
esp68*DR0*motif_fin*Rv2813c

DRb2, nor DR4, nor DR5 were found by CRASS. It is likely that CRASS does not identify DR variants. 48 spacers were found by CRASS whereas CRISPRbulider finds 47. CRASS is fully automatized whereas CRISPRbuilder allows human action (and correction). The same spacers are found, however, with CRASS, spacer 66 in counted twice with one mutation for one of the two, that is why there is one more spacer with CRASS than with CRISPRbuilder. Moreover spacer 68 has 2 mutations by CRASS relatively to the true spacer 68, whereas spacer 27 bears one mutation with CRASS. Last but not least, CRASS does produce many loops in many directions and does not reconstitute the true CRISPR locus

The string starts with spacer 2 and there is a loop with spacers 6-7-8 and after spacer 10 there are three possible choices.

Obervation : CRASS is not able, *per se* and without human intervention, to reconstruct the CRISPR locus of ERR751335

B. **Comparison between Crass and CRISPR_builder on SRR6407486 genome (L4.9) CRISPR locus reconstruction**

Crass Results

crisprtools extract -s crass.crispr correspondance

>G17SP5_Cov_18 -> 62
TAGTACGCCATCTGTGCCTCATACAGGTCCAGTGCCCT
>G17SP9_Cov_16 -> 15
CTGAGGAGAGCGAGTACTCGGGGCTGCCGTCTGCGCTG
>G17SP17_Cov_23 -> 23
TTCGTCGACCATCATTGCCATTCCCTCTCCCCACGT
>G17SP21_Cov_21 -> 28
CGCATGGACCCGGGCGAGCTGCAGATGGTCCGGGAG
>G17SP545_Cov_12 -> 20
CGGGCAGCGTTCGACACCCGCTCTAGTTGACTTCCGG
>G17SP26_Cov_33 -> 18
GCGTGAAACCGCCCCCAGCCTCGCCGGGGCCGCCTAG
>G17SP30_Cov_29 -> 52
GCCCCGTGGATGGCGGATGCGTTGTGCGCGCAAGT
>G17SP37_Cov_24 ->
42 TTTATCACTCCCGACCAAATAGGTATCGGCGTGTTCAA
>G17SP42_Cov_29 -> 4
TCGCAAGCGCCGTGCTTCCAGTGATCGCCTTCTA
>G17SP47_Cov_26 -> 38
TGCCCCGGCGTTTAGCGATCACAACACCAACTAATG
>G17SP487_Cov_21 -> 29
TGGATTGCGCTAACTGGCTTGGCGCTGATCCTGGTG
>G17SP60_Cov_7 -> 25 troncated
CTTGAATAACGCGCAGTGAATTTCG
>G17SP69_Cov_18 -> 21
CAGGTGAGCAACGGCGGCGGCCCCCTGGCGGCCACGGGTCG
>G17SP73_Cov_18 -> 22
ATGGGATATCTGCTGCCCGCCCGGGGAGATGCTGTCCGAG
>G17SP77_Cov_16 -> 3
TTTTTGCCTCATGCTTGGGCGACAGCTTTTGACCAA
>G17SP1302_Cov_6 -> 34
CCAACCTCACCGCCTGCTGGGTGAGACGTGCTCGCCGCGA
>G17SP88_Cov_24 -> 33
CCTCAGCTCAGCATCGCTGATGCGGTCCAGCTCGTCCGT
> G17SP93_Cov_20 -> 24
TTGCGCCAACCCTTTCGGTGTGATGCGGATGGTCGGCTCGG
>G17SP97_Cov_12 -> 66
GCATATCGCCCGCCACACCACAGCCACGCTACTGCTCCAT
>G17SP101_Cov_21 -> 53
CCGACGATGGCCAGTAAATCGGCGTGGGTAACCGATCCGG
>G17SP105_Cov_17 -> 41
AGGTTCGCGTCAGACAGGTTCGCGTCGATCAAGTCCG

>G17SP110_Cov_14 -> 68
TTGAACCGCCCTTCGCGCGGTGTTTCGGCCGTGCCCGA
>G17SP114_Cov_26 -> 14
ACCAATGCGTCGTCATTTCCGGCTTCAATTTCAGCCT
>G17SP123_Cov_22 -> 26
ATTCGCACGAGTTCCCGTCAGCGTCGTAAATCGCCA
>G17SP120_Cov_17 -> 13
TTTTCTGACCACTTGTGCGGGATTAGCGGGCTTAG
>G17SP141_Cov_23 -> 63
CTGACGGCACGGAGCTTTCCGGCTTCTATCAGGTA
>G17SP146_Cov_25 -> 19
ACTCGGAATCCCATGTGCTGACAGCGGATTCGCAT
>G17SP155_Cov_21 -> 51
ATTTTGAGCGCGAACTCGTCCACAGTCCCCCTTTCAG
>G17SP161_Cov_33 -> 39
CAGCGAAATACAGGCTCCACGACACGACCACAACGC
>G17SP222_Cov_20 -> 12
AACACCTCAGTAGCACGTCATACGCCGACCAATCATCAG
>G17SP172_Cov_16 -> 1
TTAAAACCGTGTTGCACTGCAACCCGGAATTCTTGCAC
>G17SP201_Cov_17 -> 2
CATAGAGGGTCGCCGGCTCTGGATCACGCTCCCCTAGTCGT
>G17SP177_Cov_15 -> 27
CCGGCAACAATCGCGCCGGCCCGCGCGGATGACTCCG
>G17SP197_Cov_14 -> 65
TGGACGCAGAATCGCACCGGGTGCGGGAGGTGCAGCA
>G17SP208_Cov_27 -> 64
CCTCATGGTGGGACATGGACGAGCGCGACTATCGGG
>G17SP215_Cov_16 -> 40
TCTTGACGATGCGGTTGCCCCGCGCCCTTTTCCAGCC
>G17SP472_Cov_23 -> 32
TTGGAGCGTGTCACCGCAGACGGCACGATTGAGACAA
>G17SP798_Cov_8 -> 67
ACACCGCCGATGACAGCTATGTCCGAGTGACATCCTCCCA

SRR6407486. (CRISPR builder)

Rv2816c*motif_debut1*DR0*esp1*DR0*esp2*DR0*esp3*DR0*esp4*DR0*esp12*DR0*esp13*DR0*esp14*DR0*esp15*DR0*esp18*DR0*esp19*DR0*esp20*DR0*esp21*DR0*esp22*DR0*esp23*DR0*esp24*DR0*esp25*DRb2*esp26*DR0*esp27*DR0*esp28*DR0*esp29*DR0*esp32*DR0*esp33*DR0*esp34*rDRa1*IS6110*DRb1*esp35*DR0*esp36*DR0*esp37*DR0*esp38*DR0*esp39*DR0*esp40*DR0*esp41*DR0*esp42*DR0*esp51*DR0*esp52*DR0*esp53*DR0*esp62*DR0*esp63*DR0*esp64*DR6*esp65*DR0*esp66*DR4*esp67*DR5*esp68*DR0*motif_fin*Rv2813c*

Further observations

Spacer blocks 35,36,37 are missed by Crass (may be because of the IS6110 insertion). No information relative to IS6110 are given. The reconstruction looks more linear than for the former studied genome, however spacer 68 does appear alone and not connected to spacer 67 (see Figure) . Concerning neighboring sequences of the CRISPR Locus, the end motif is found, however the remaining does not look very robust.

>G17FL434 -> esp15 ...
CTGAGGAGAGCGAGTACTCGGGGCTGCCGTCTGCGCTGGTCGTCAGACCCAAAA
CCCCAGATCGGAAGAGCACACG
>G17FL1069 -> esp4 ...
TCGCAAGCGCCGTGCTTCCAGTGATCGCCTTCTAGTCGTCAGACCCAAAACCCCG
AGAGGGGACGGAGATCGGAAGA
>G17FL1590 -> esp66 ...
GCATATCGCCCGCCACACCACAGCCACGCTACTGCTCCATGTCCTCAGACCCAAG
ATCGGAAGAGCAC
>G17FL1188 -> esp63 ...
CTGACGGCACGGAGCTTTCCGGCTTCTATCAGGTAGTCGTCAGACCCAAAACCCC
GAGAGGGAGATCGGAAG
>G17FL1544 -> esp1 ...
TTAAAACCGTGTTGCACTGCAACCCGGAATTCTTGCACGTCGTCAGACCCAAAAC
CCCGAGAGATCGGAAGAGC
>G17FL1018 -> end_motif ...
TACGACGACTGGGTCGCCTCCGCGTCTGTTGACCGGCATTCAGGATGAGCATGAT
GGCGGCGTTGA
>G17FL285 -> esp32
TTGGAGCGTGTCACCGCAGACGGCACGATTGAGACAAGTCGTCAGACCAGATCG
GAAGAGCACA

A: ERR751335 (M. africanum; Lineage 5)

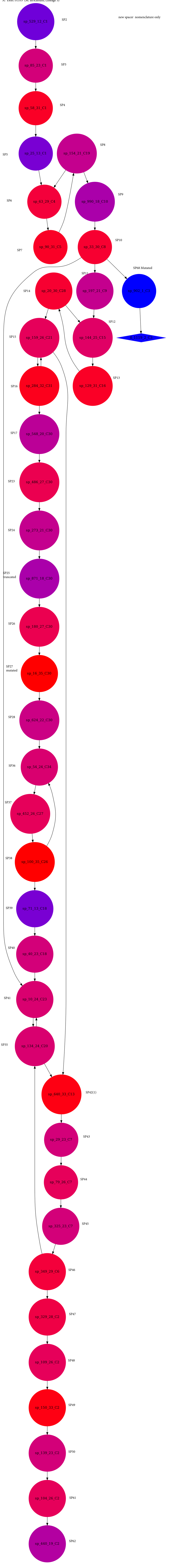

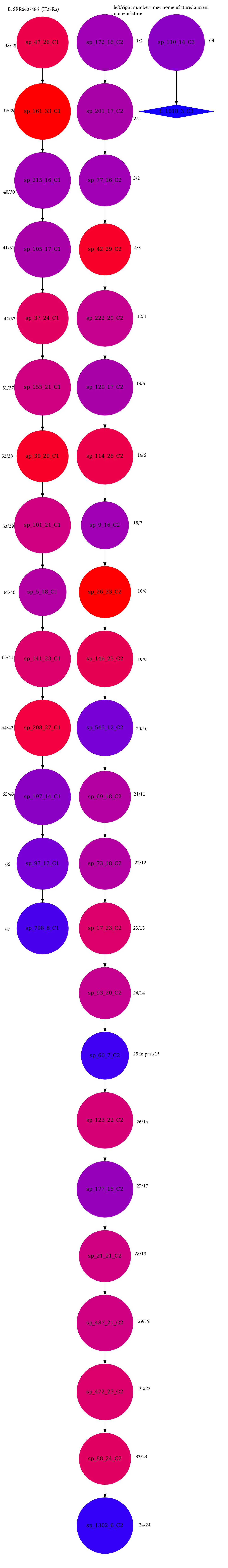
B: SRR6407486 (H37Ra)
left/right number : new nomenclature/ ancient nomenclature
38/28
sp_47_26_C1
39/29
sp_161_33_C1
40/30
sp_215_16_C1
41/31
sp_105_17_C1
42/32
sp_37_24_C1
51/37
sp_155_21_C1
52/38
sp_30_29_C1
53/39
sp_101_21_C1
62/40
sp_5_18_C1
63/41
sp_141_23_C1
64/42
sp_208_27_C1
65/43
sp_197_14_C1
66
sp_97_12_C1
67
sp_798_8_C1
sp_172_16_C2
1/2
sp_201_17_C2
2/1
sp_77_16_C2
3/2
sp_42_29_C2
4/3
sp_222_20_C2
12/4
sp_120_17_C2
13/5
sp_114_26_C2
14/6
sp_9_16_C2
15/7
sp_26_33_C2
18/8
sp_146_25_C2
19/9
sp_545_12_C2
20/10
sp_69_18_C2
21/11
sp_73_18_C2
22/12
sp_17_23_C2
23/13
sp_93_20_C2
24/14
sp_60_7_C2
25 in part/15
sp_123_22_C2
26/16
sp_177_15_C2
27/17
sp_21_21_C2
28/18
sp_487_21_C2
29/19
sp_472_23_C2
32/22
sp_88_24_C2
33/23
sp_1302_6_C2
34/24
sp_110_14_C3
68
fl_1018_3_C3
