# Supplementary material for: CRISPRbuilder-TB: “CRISPR-builder for tuberculosis”. Exhaustive reconstruction of the CRISPR locus in mycobacterium tuberculosis complex using SRA
Source: PLoS Comput Biol. 2021 Mar 5;17(3):e1008500. doi: 10.1371/journal.pcbi.1008500 (PMC7968741; doi:10.1371/journal.pcbi.1008500)
Supplement: S3 Text — (PDF) [file pcbi.1008500.s011.pdf]

S3 Text Algorithms used to reconstruct CRISPR locus (spacer discovery, and contiguage)

Algorithm 1: Python-like pseudo-code of new (variant of) spacer discovery

```
#Set of known spacers
known_spacers = {i: [sequence(i) for sequence(i) in Van Embden],
                 for i = 1..98}
putative_spacers = []
for each g in genomes:
    for each r in g.reads:
        if s subword of r matches DR0[-12:]([ACGT]{10,70})DR0[:12]:
        # some read matches the regular expression
        sp = s[12:-12]
        if sp not in known_spacers.values():
            putative_spacers.append(sp)
considered_sequences = [seq for seq in set(putative_spacers)
                        if putative_spacers.count(seq) > 50]
for sp in considered_sequences:
    if max([similarity(sp, known_spacers[i][0]) for i in 1..98]) < 0.95:
        # A new spacer is discovered
            known_spacers[len(known_spacers)+1] = [sp]
    else:
            # id number of the similar spacer
            id = idxmax([similarity(sp, known_spacers[i][0]) for i in 
1..98])
            known_spacers[id].append(sp)
```

Algorithm 2: Python-like pseudo-code of contiguage

```
seqs = blastn(sequences_of_interest, reads, evalue = 1e-7)
# k-merization of reads of interest
n = len(seqs[0])
k = int(4*n/5)
seqs = [[seq[u:u+k] for u in 0..n-k] for seq in seqs]
# Contiguage
contigs = []
while len(seqs) > 0:
    contig = choice(seqs)
    seqs.remove(contig)
    forward = True
    while forward:
        # kmers that begin like the end of the contig
        kmers = [seq for seq in seqs if seq[:-1] == contig[-n+1:]]
        for kmer in kmers:
            seqs.remove(kmer)
        # A nucleotide is more frequent at the end of the kmers ?
        frequences = {u: [[kmer[-1] for kmer in kmers].count(u) for u in 
"ACGT"}
        occurrences = sorted(frequences.values())
        if occurrences[0] > 3*occurrences[1]:
            # Adding the most frequent ending nucleotide to the contig end
            new_nucl = idxmax(frequences.values())
            contig += new_nucl
        else:
            forward = False
```

```
    while not forward:
        # kmers that end like the beginning of the contig
        kmers = [seq for seq in seqs if seq[1:] == contig[:n-1]]
        for kmer in kmers:
            seqs.remove(kmer)
        # A nucleotide is more frequent at the beginning of the kmers ?
        frequences = {u: [[kmer[0] for kmer in kmers].count(u) for u in
“ACGT”}
        occurrences = sorted(frequences.values())
        if occurrences[0] > 3*occurrences[1]:
            # Adding the most frequent beginning nucleotide to the contig
head
            new_nucl = idxmax(frequences.values())
            contig = new_nucl+contig
        else:
            contigs.append(contig)
```
