# Supplementary material for: CRISPRbuilder-TB: “CRISPR-builder for tuberculosis”. Exhaustive reconstruction of the CRISPR locus in mycobacterium tuberculosis complex using SRA
Source: PLoS Comput Biol. 2021 Mar 5;17(3):e1008500. doi: 10.1371/journal.pcbi.1008500 (PMC7968741; doi:10.1371/journal.pcbi.1008500)
Supplement: S1 Fig — (PPTX) [file pcbi.1008500.s012.pptx]

## Slide 1
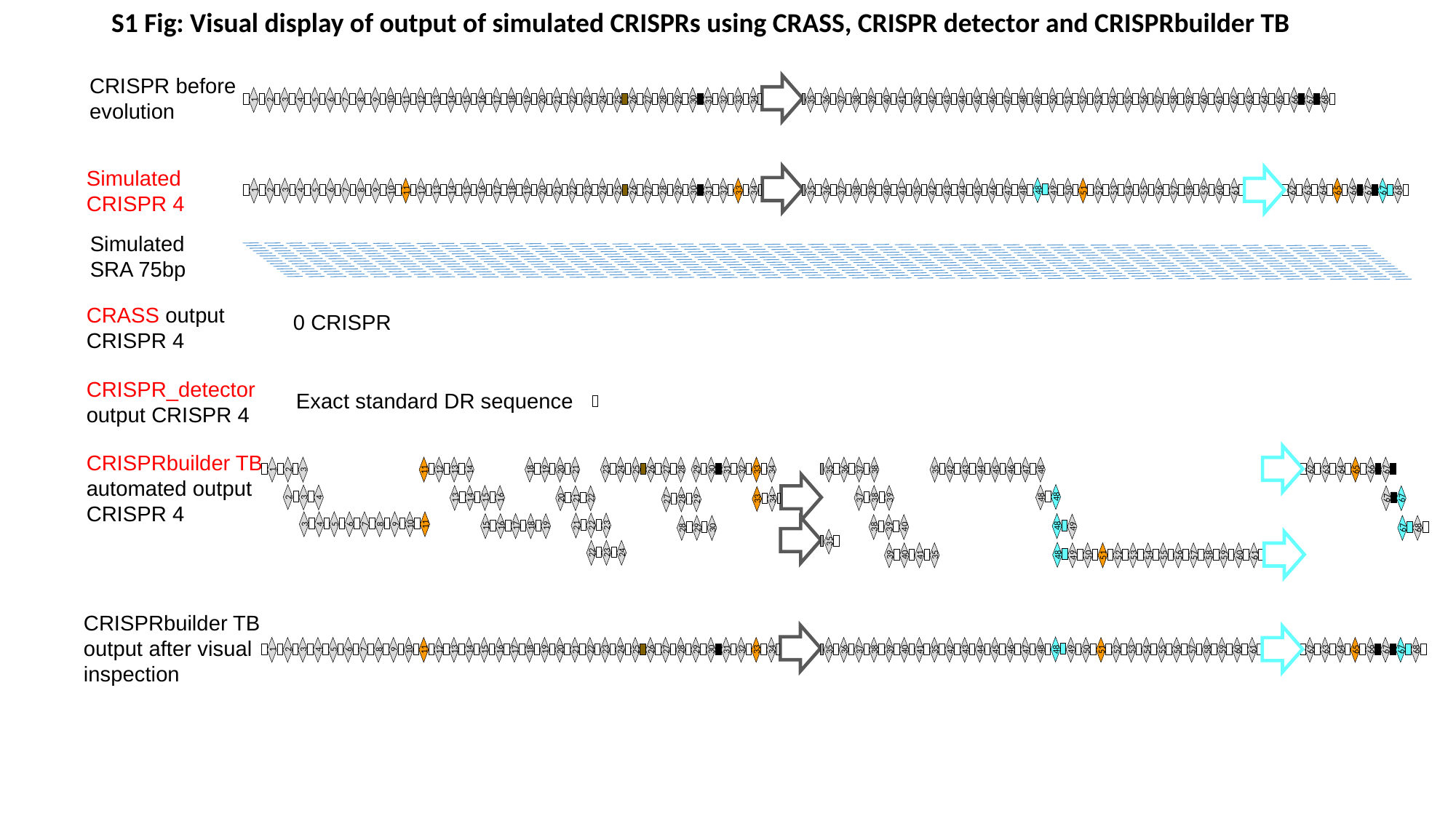

S1 Fig: Visual display of output of simulated CRISPRs using CRASS, CRISPR detector and CRISPRbuilder TB
CRISPR before evolution
1
2
3
4
5
6
7
8
9
10
11
12
13
14
15
16
17
18
19
20
21
22
23
24
25
26
27
28
29
30
31
32
33
34
35
36
37
38
39
40
41
35
42
43
44
45
46
47
48
49
50
51
52
53
54
55
56
57
58
59
60
61
62
63
64
65
66
67
68
Simulated CRISPR 4
48
1
2
3
4
5
6
7
8
9
10
11
12
13
14
15
16
17
18
19
20
21
22
23
24
25
26
27
28
29
30
31
32
33
34
35
36
37
38
39
40
41
35
42
43
44
45
46
47
48
49
50
51
52
53
54
55
56
57
58
59
60
61
62
63
64
65
66
67
68
67
Simulated SRA 75bp
CRASS output CRISPR 4
0 CRISPR
CRISPR_detector output CRISPR 4
Exact standard DR sequence
CRISPRbuilder TB automated output CRISPR 4
1
2
3
11
12
13
14
18
19
20
21
23
24
25
26
27
28
29
30
31
32
33
34
35
36
37
38
35
42
43
44
45
46
47
48
62
63
64
65
66
67
48
48
2
3
4
13
14
15
16
37
38
39
67
67
20
21
22
33
34
27
28
29
3
4
5
6
7
8
9
10
11
21
22
23
15
16
17
18
19
48
49
38
39
40
28
29
30
68
67
35
48
49
50
51
52
53
54
55
56
57
58
59
60
61
22
23
24
39
40
41
35
CRISPRbuilder TB output after visual inspection
48
1
2
3
4
5
6
7
8
9
10
11
12
13
14
15
16
17
18
19
20
21
22
23
24
25
26
27
28
29
30
31
32
33
34
35
36
37
38
39
40
41
35
42
43
44
45
46
47
48
49
50
51
52
53
54
55
56
57
58
59
60
61
62
63
64
65
66
67
68
67

## Slide 2
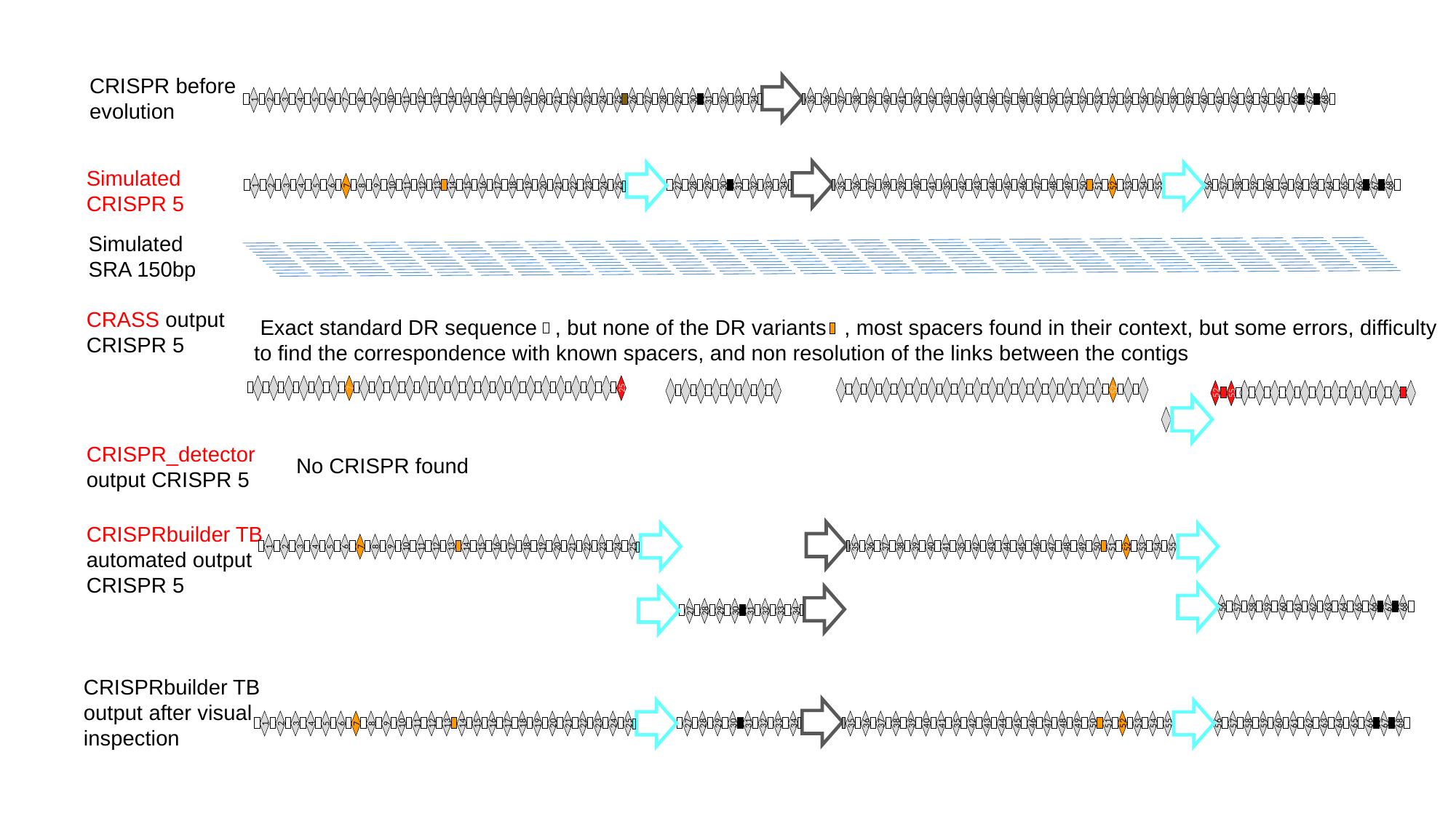

CRISPR before evolution
1
2
3
4
5
6
7
8
9
10
11
12
13
14
15
16
17
18
19
20
21
22
23
24
25
26
27
28
29
30
31
32
33
34
35
36
37
38
39
40
41
35
42
43
44
45
46
47
48
49
50
51
52
53
54
55
56
57
58
59
60
61
62
63
64
65
66
67
68
Simulated CRISPR 5
1
2
3
4
5
6
7
8
9
10
11
12
13
14
15
16
17
18
19
20
21
22
23
24
25
27
28
29
30
31
32
33
34
35
36
37
38
39
40
41
35
42
43
44
45
46
47
48
49
50
51
52
53
54
55
56
57
58
59
60
61
62
63
64
65
66
67
68
Simulated SRA 150bp
CRASS output CRISPR 5
 Exact standard DR sequence , but none of the DR variants , most spacers found in their context, but some errors, difficulty to find the correspondence with known spacers, and non resolution of the links between the contigs
1
2
3
4
5
6
7
8
9
10
11
12
13
14
15
16
17
18
19
20
21
22
23
24
25
35
36
37
38
39
40
41
35
42
43
44
45
46
47
48
49
50
51
52
53
54
26
27
28
29
30
31
32
33
56
57
58
59
60
61
62
63
64
65
66
67
52
55
55
CRISPR_detector output CRISPR 5
No CRISPR found
CRISPRbuilder TB automated output CRISPR 5
1
2
3
4
5
6
7
8
9
10
11
12
13
14
15
16
17
18
19
20
21
22
23
24
25
35
36
37
38
39
40
41
35
42
43
44
45
46
47
48
49
50
51
52
53
54
55
56
57
58
59
60
61
62
63
64
65
66
67
68
27
28
29
30
31
32
33
34
CRISPRbuilder TB output after visual inspection
1
2
3
4
5
6
7
8
9
10
11
12
13
14
15
16
17
18
19
20
21
22
23
24
25
27
28
29
30
31
32
33
34
35
36
37
38
39
40
41
35
42
43
44
45
46
47
48
49
50
51
52
53
54
55
56
57
58
59
60
61
62
63
64
65
66
67
68

## Slide 3
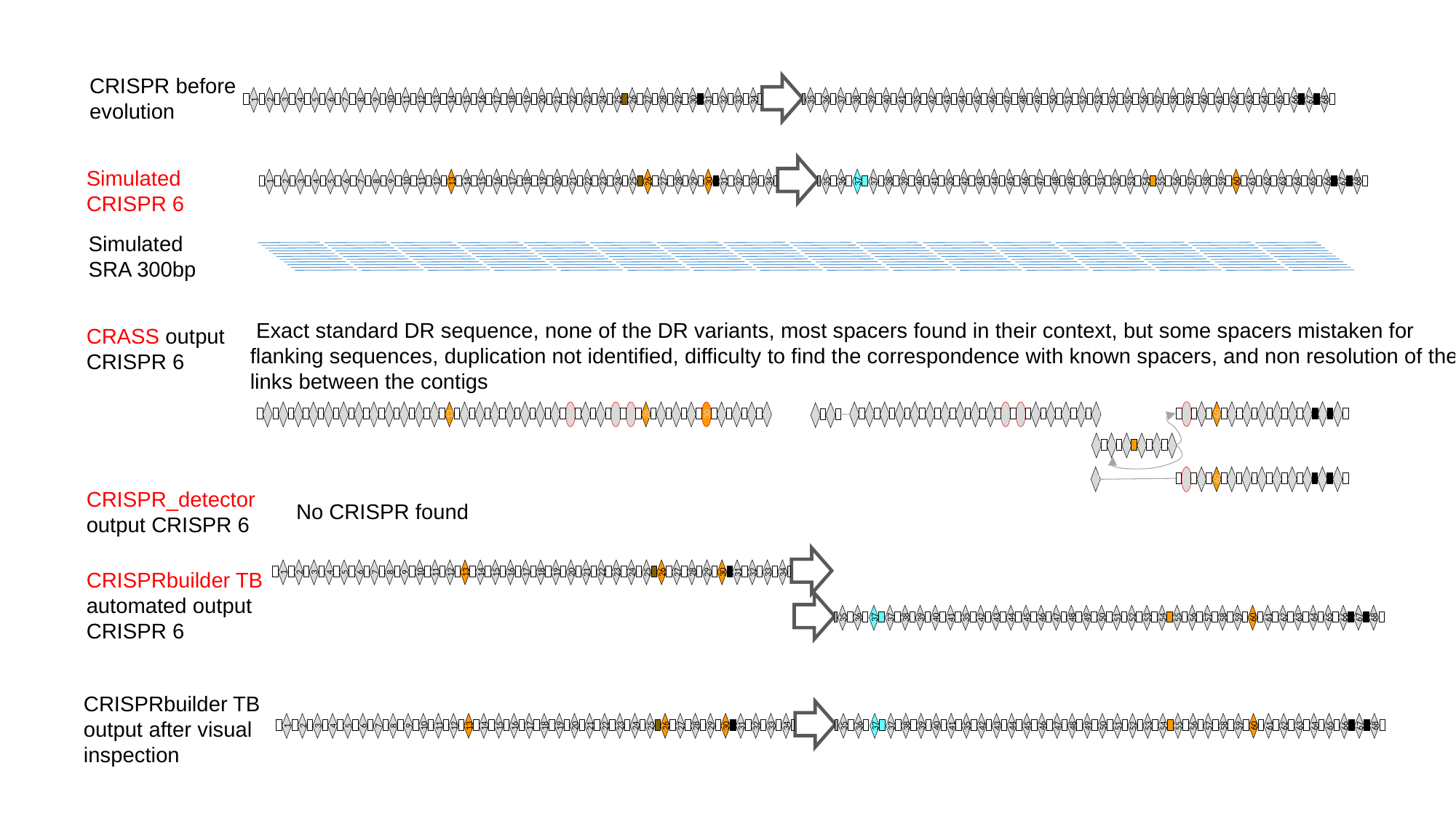

CRISPR before evolution
1
2
3
4
5
6
7
8
9
10
11
12
13
14
15
16
17
18
19
20
21
22
23
24
25
26
27
28
29
30
31
32
33
34
35
36
37
38
39
40
41
35
42
43
44
45
46
47
48
49
50
51
52
53
54
55
56
57
58
59
60
61
62
63
64
65
66
67
68
Simulated CRISPR 6
1
2
3
4
5
6
7
8
9
10
11
12
13
14
15
16
17
18
19
20
21
22
23
24
25
26
27
28
29
30
31
32
33
34
35
36
37
37
38
39
40
41
35
42
43
44
45
46
47
48
49
50
51
52
53
54
55
56
57
58
59
60
61
62
63
64
65
66
67
68
Simulated SRA 300bp
 Exact standard DR sequence, none of the DR variants, most spacers found in their context, but some spacers mistaken for flanking sequences, duplication not identified, difficulty to find the correspondence with known spacers, and non resolution of the links between the contigs
CRASS output CRISPR 6
58
59
60
61
62
63
64
65
66
67
68
1
2
3
4
5
6
7
8
9
10
11
12
13
14
15
16
17
18
19
20
21
22
23
24
25
26
27
28
29
30
31
32
33
34
37
38
39
40
41
35
42
43
44
45
46
47
48
49
50
51
52
35
36
52
53
54
55
56
57
52
58
59
60
61
62
63
64
65
66
67
68
CRISPR_detector output CRISPR 6
No CRISPR found
1
2
3
4
5
6
7
8
9
10
11
12
13
14
15
16
17
18
19
20
21
22
23
24
25
26
27
28
29
30
31
32
33
34
CRISPRbuilder TB automated output CRISPR 6
35
36
37
37
38
39
40
41
35
42
43
44
45
46
47
48
49
50
51
52
53
54
55
56
57
58
59
60
61
62
63
64
65
66
67
68
CRISPRbuilder TB output after visual inspection
1
2
3
4
5
6
7
8
9
10
11
12
13
14
15
16
17
18
19
20
21
22
23
24
25
26
27
28
29
30
31
32
33
34
35
36
37
37
38
39
40
41
35
42
43
44
45
46
47
48
49
50
51
52
53
54
55
56
57
58
59
60
61
62
63
64
65
66
67
68
