# Supplementary material for: CRISPRbuilder-TB: “CRISPR-builder for tuberculosis”. Exhaustive reconstruction of the CRISPR locus in mycobacterium tuberculosis complex using SRA
Source: PLoS Comput Biol. 2021 Mar 5;17(3):e1008500. doi: 10.1371/journal.pcbi.1008500 (PMC7968741; doi:10.1371/journal.pcbi.1008500)
Supplement: Attachment — Submitted filename: Answer to reviewers Plos Comp_Sept2020.docx [file pcbi.1008500.s013.docx]

Answer to reviewers Plos Comp. 2020

Reviewer #1: The work presented by Guyeux and co-authors in this manuscript focuses on reconstructing the CRISPR locus of Mycobacterium tuberculosis complex strains from short reads sequences directly. The authors developed a bioinformatics pipeline to achieve this goal and presented their results compared to those obtained from other similar tools. A major strength of their work is that the performance of their pipeline (named CRISPRbuilder-TB) is evaluated by reconstructing CRISPR loci of simulated and complete MTBC genomes, where the structure of CRISPR loci are known. They show that 1/3 of assembled genomes contain mis-assemblies and erroneous CRISPR loci, and thus advocate for the use of their pipeline to reconstruct CRISPR loci from short reads directly. They also show how existing tools do not capture the full diversity at the CRISPR locus, including duplications, spacer and direct repeat variants, and locations of IS6110 insertions.

Major comments:

- The authors should include documentation on their project GitHub page (<https://github.com/cguyeux/pyMTC/>) explaining how the pipeline can be downloaded and run locally by providing examples, in addition to any software requirements.

-> the whole interface has been reshaped and moved to <https://github.com/cguyeux/CRISPRbuilder-TB> to fulfill all requirements.

- The authors provide a comprehensive list of available CRISPR identification tools. However, they only compared their tool (CRISPRbuilder-TB) to two other independent tools (Table 1). It is not cleat to this reviewer why only Crass and CRISPRdetector were used as comparison tools, whereas other cited CRISPR predictor tools (CRT, CrisprFinder, PILER-CR, CRISPRCasFinder, CRTmeta or metaCRISPR) were not. As some of these tools only accept assembled genomes as input, simulated reads could be assembled first and then assemblies used to run the remaining CRISPR prediction tools.
-> It is true that complete genome assembly could be performed before running tools such as CRT, CrisprFinder, PILER-CR, CRISPRCasFinder. However, this kind of process requires other expertise than that required for most common analyses: the tools made available to infer resistance and position in global MTC phylogeny (Phyresse, Snippy, etc.) only use SRA without genome reconstruction. Hence, most of the community of clinicians is not used to genome reconstruction. And the people who try often make mistakes as seen in the public databases genomes (~1/3 having H37Rv CRISPR loci instead of their own). The logic we followed is the same as Crass and CRISPRdetector : our aim is to provide the equivalent of Phyresse for TB-CRISPR, *i.e.* to enable CRISPR-derived data using a single tool starting from SRA. We clarified this objective at several locations in the text :

-Abstract line 38, we added « directly »

-Author summary line 54, we added « without requiring genome assembly »

-line 120, we added : « without any genome assembly step »

-line 296, we added : « This tool is in the line of tools avoiding genome assembly to reduce computational time and potential biases such as Phyresse, Snippy, etc. [27]. »

- Both in Table 1 and Table 2 is not obvious from the text provided that reconstructed CRISPR loci by CRISPRbuilder-TB are identical to simulated loci (Table 1) or reference genome loci (Table 2). A simple visual representation of simulated and reference vs. reconstructed CRISPR loci is needed to easily identify and compare regions of homology, order of CRISPR features (DR, spacers) and variants (SNPs, duplications, IS6110) between simulated and reference compared to reconstructed loci.

-> As the Table was quite dense already, we chose to provide Supplementary files (3 to 5) that are fully explicit. This in addition increases the number of interpreted reconstructions, confirming the previous results. In addition, we tried to clarify the content of the text in the Table to make it clear that only CRISPRbuilder provided an exhaustive and correct information : we made explicit that CRISPRbuilder output was correct by precising « the 100% *correctly* decoded sequence », we made explicit that CRISPRdetector output was very limited when present by adding « Confirmation of » 1 CRISPR. And « Correct sequence of » DR0 but none of the DR variants identified.

- Although, the authors have published a separate study on the evolution of CRISPR locus on MTBC, they should discuss whether there is evolutionary evidence that the CRISPR locus is functional. According to <https://www.biorxiv.org/content/10.1101/2019.12.13.875765v1> , the CRISPR locus “of M. tuberculosis did not evolve by classical CRISPR adaptation (incorporation of new spacers) since the last most recent common ancestor of virulent lineages.” Would the authors interpret these results as a clear indication of a non-functional CRISPR locus?
–> It is difficult with this study to be formal about any functionality of CRISPR locus in MTC. We can say that the combination of tuberculosis life style and CRISPR features makes the locus inefficient for acquiring new spacers as now extensively shown by CRISPR TB diversity. But to infer that non-functionality of CRISPR is responsible for this situation requires independent *in vitro* testing. We added two comments in the conclusion to clarify this point and mention possible functional consequences. “but no insertion of new spacers » (line 496) and « discussing for instance the likely non-functionality of spacer acquisition of this locus» (line 499-500)

Minor comments:

Abstract:

In ‘compared to more generalist tools’, please indicate here what these tools are here in the abstract.

Authors summary:

Line 55: ‘using short read sequences’ not ‘Short Reads Archives’ 🡪 done
Line 56: ‘current assembled genomes’ 🡪 done
Line 60: list here what existing methods CRISPRbuilder-TB was compared to 🡪 done

Introduction:

Line 67: indicate in brackets and include citations of the first two Mtb sequenced isolates 🡪 done.
Line 73: given that spoligotyping is not taxonomically precise or fully ‘correct’, please replace ‘allows a correct taxonomical assignment’ with ‘allows an approximate taxonomical assignment’ 🡪 done

Line 89: in more than a hundred thousand samples. Also include citation at the end of this statement. 🡪 we wrote « one hundred » as suggested by reviewer 2 and added a reference.

Line 97: they have the same limitations as in vitro spoligotyping techniques. 🡪 done

Line 99. Use WGS abbreviation instead of ‘whole genome sequencing’, here and elsewhere in the text. 🡪 done

Line 99. By traditional de novo assembly tools 🡪 done


Results:

a. Description of CRISPRbuilder-TB

Line 129. A known limitation 🡪 done

Figure 2. In Dataset #1 Validation sample, do the authors used 7 complete (sequenced to completion) reference genomes? If so, they may want to change ‘Assembled genomes
collection from NCBI’ to ‘Complete genomes collection from NCBI’ 🡪 done

In lines 130 to 132. A more explicit description on how CRISPR locus associated reads are extracted is needed here. From Figure 1 step 1, DR sequences are used to fish out CRISPR locus associated sequences from short sequence reads. 🡪 done (actually not only the DRs are used to fish out useful reads but also any other remarkable sequences. This was made more explicit in the text. « *We kept not only reads similar to DR sequences but also reads similar to other sequences that may be linked to the CRISPR (Figure 1 step 1) »* and we further clarified the that the expression of the composition of the CRISPR is a second aim of this catalogue : «  *This catalogue is thereafter also aimed to express the composition of a definite CRISPR in a self-explanatory way* *»*

Figure 1 illustrates very well CRISPRbuilder-TB pipeline but lacks a legend in the manuscript. Figure 1 would also benefit from including what bioinformatic tools were used at each step. 🡪 done. The following legend was added : « *All steps were performed using tailored python scripts embedded in CRISPRbuilder-TB. Step 1: WGS reads filtering by blasting the reads with the catalog of remarkable sequences (threshold e-value=1e-7). Step 2: transformation of reads into k-mers which length was adjusted to read length n (k-mer=4/5n). Step 3: De Bruijn graph reconstruction implemented in python (see Supplementary File 9, part b). Step 4: Replacement of known sequences by their annotation as per the catalogue of sequences of interest. Rare unknown sequences are kept as such for manual inspection. Step 5: manual reconstruction of full CRISPRs using the available contigs (mostly concatenation according to IS6110 recovered ends)* »

Line 138. It would be helpful to report how many SNP differences (mismatches) the authors find among variants of the same spacer. This would inform the maximum number of mismatches in silico spoligotyping tools use to search for spacer occurrences in SRA data.

🡪 The information was hidden in Supplementary file 2 ; we added color on the sequences to highlight the SNPs and we added some text in the ms to make this explicit. (« *; max=2 SNPs per spacer,* ***Supplementary File 2*** »), and we took the oportunity to add the equivalent for DRs « *max SNPs per DR =3* ».

Lines 148 – 149. What do the authors mean by ‘translate these contigs in strings of words’? Do they refer to annotating contigs with CRISPR locus features such as DR, spaces, etc. If so the authors may want to use the term ‘annotation’ and rename step4 in Figure 1 ‘Translation into explicit contigs’ to ‘CRISPR annotation of contigs’. (answer see below)
Line 151. Related to the point above, the authors may want to use the term ‘Unannotated regions’ of contigs instead of ‘untranslated parts’.
🡪 In our understanding, annotation corresponds to the mention of sequences of interest when they exist. Here the idea is to keep any information that is not understood: initial information is replaced by its corresponding annotation if it exists. Otherwise it is kept as such. This is why we initially chose the word translate. We rephrased to circumvent potential misunderstanding associated with the word « translate » : « *The specificity of CRISPRbuilder-TB is then to replace known subsequences into strings of words using the MTC CRISPR remarkable sequences catalog described above* *(Figure 1 step 4). This replacement allows fast manual inspection of the contigs, highlithing potentially compatible contig ends and unknown sequences* »

b. Reconstruction of simulated CRISPR

Table 1. It is not cleat to this reviewer why only Crass and CRISPRdetector were used as comparison tools, whereas other cited CRISPR predictor tools (CRT, CrisprFinder, PILER-CR, CRISPRCasFinder, CRTmeta or metaCRISPR) were not. As some of these tools only accept assembled genomes, simulated reads could be assembled first and then assemblies used to run the remaining CRISPR prediction tools.
-> As already explained above, it is true that complete genome assembly could be performed before running tools such as CRT, CrisprFinder, PILER-CR, CRISPRCasFinder. However, this kind of process requires other expertise than that required for most common analyses. The logic we followed is the same as Crass and CRISPRdetector : our aim is to provide the equivalent of Phyresse for TB-CRISPR, *i.e.* to enable CRISPR-derived data using a single tool starting from SRA.
Table 1. As pointed before, it is preferable to use the term ‘non-annotated contig sequences’ than ‘untranslated sequences’
🡪done. As explained above, we described these sequences as « unknown ».

Line 163. Indicate how short (in bp) the shortest reads used by CRISPRbuilder were.
🡪 done (75bp)

Line 163. As pointed before, use ‘annotation’ instead of ‘translation’.
🡪 done

Evaluation of CRISPR locus reconstruction based on WGS data of MTC reference strains

Line 187. The authors should stress here that the seven reference strains had been sequenced to completion, that is, they had an ungapped and complete chromosome sequence resolved. This is somehow implied with the term ‘reference’ genome.

🡪 done. We made this explicit in the title « *c. Evaluation of CRISPR locus reconstruction based on WGS data of MTC reference strains with complete genome sequences.* » and rephrased the first sentence accordingly; « *the seven best MTC studied strains for which complete genome sequence is available, using corresponding sequencing runs.* ». We also amended Table 2 title accordingly : « ***Table 2 – CRISPR-Cas features of reference strains according to complete genome sequences and according to CRISPR-builder run on WGS-data*** », as well as table section line 189 (« *CRISPR features derived from complete genome sequences using Spolpred-like tool*  »).

Line 203. Do the authors mean CRISPRbuilder-TB reconstructed sequences were identical to those of the complete reference genome? Or those assembled de novo from short reads? Same point in Line 212 ‘assembled genome’.

🡪 again corrected for « complete genome sequences », as well as in other parts of the manuscript.

Line 207. Citation needed right after statement ‘as described previously’.
🡪 done

219. Do the authors mean ‘public reference genomes’ by ‘public assemblies’ here? As pointed before, it is important to differentiate between complete reference genomes (ungapped, resolved and annotated) vs. de novo assemblies (made up of contigs).
🡪 again corrected for « complete genome sequences ».

d. CRISPR region in other MTC assembled genomes.

Line 230. In the statement ‘Most sequences seemed of high quality.’, what quality control metrics did the authors used to assess the quality of these 187 MTC public genomes? What sampling strategy was used to select these 187 MTC genomes? Are these complete genomes or assembled?
🡪 again corrected for « complete genome sequences ». There was no selection (we clarified this in the text « *we extracted the CRISPR-locus from all the additional MTC complete genomes we identified*  »


e. CRISPR evolutionary events in MTC

As pointed before, for line 138. It would be helpful to report how many SNP differences (mismatches) the authors find among variants of the same spacer. This would inform the maximum number of mismatches in silico spoligotyping tools use to search for spacer occurrences in SRA data.

🡪 done. We added the following sentence line 289-290 : «  Of note, the spacer variants had up to 2 SNPs compared to reference sequence.  »

f. Computational speed

Discussion.

Line 323. Use ‘complete reference genomes’ instead of ‘reference samples’. 🡪 done.
Line 325. Use ‘concordant with complete genome’ instead of ‘concordant with assembled ones’🡪 done.

4. Material and methods
a. Sequence data

Line 480 to 481, replace ‘both assembled genomes and short reads sequencing’ with ‘both complete genomes and short reads sequences”.🡪 done.

Lines 486 and 487. What selection criteria did the authors followed to select these assembled genomes from public repositories? 🡪 no selection as explained above. This was clarified in the text. « *A second set of data concerns all non-reference clinical isolates for which complete genomes were available at the time of the study (but for which no short reads sequences were available)*. »

Line 489. Do the authors mean European Nucleotide Archive (ENA) by EMBL-SRA? 🡪 yes, this was made more explicit in the text : « *sequence reads archives from NCBI and from European Nucleotide Archive (ENA)* »

e. Locus reconstruction
Contiguage

It is not clear to this reviewer why the authors did not make use of an established de novo assembler (such as Velvet or Spades) to assembly selected reads and, instead, came up with their own algorithm.
 🡪Calling for more versatile programs and include adjusted parameters is necessarily more computationnally intensive than implementing a directly tailored tool. As we had the know-how to build an independent package, and as this did not impact the user interface, we preferred to choose this option. We made adjustements and verification to provide a fully reliable, computationally light tool.

f. MTC CRISPR simulation

Lines 708 – 712. Can the authors specify what bioinformatics tool they used to simulate short reads? 🡪 The method was made explicit : «  using a homemade python program using random extraction of sequences of a definite length *l* from a random position i until desired coverage is reached (items extraction from a list [i : i+*l*+1] ) » (lines 743-745)

Reviewer #2: This is a well written paper that provides important novel insight in the CRISPR genetic diversity and organisation of the locus and will help refine the MTC global population structure. It presents a detailed and comprehensive rational, approach and data for the problem. In my opinion, this is an important and worth while tool that should complement the current apps used for refining MTC phylogeny.

I have a few minor points for the authors to address.

Line 39: put comma after pipeline 🡪 done.
Line 43: replace generalist with generalised🡪 actually, we really mean generalist in the sense that these other tools are suited for a variety of species as opposed to our tool that is specific for *M. tuberculosis* complex.
Line 89: replace hundred with one hundred 🡪 done
Line 90: replace to explore with exploration of🡪 done
Line 124-126: this does not make sense. Please re-write 🡪corrected for « We emphasize its usefulness by showing that close to 50% of MTC complete genomes available in public NCBI database exhibit unreliable CRISPRs »
Line 159: replace Tool's with Tool🡪 done
Line 180: Why is the data not shown? Please explain and provide data in suppl figs. 🡪 The mutation rate that was implemented in this experiment was unreasonably high, so that many sequences did not correspond to the ones in the catalog. Replacement of sequences by their annotation could not occur. As a consequence, the recognition of corresponding ends required manual inspection like the example shown in Fig. 1 (where IS_beg can be joined with IS_end). We judged that this specific part was of little relevance and interest and chose rather to include a supplemental file showing full simulations and reconstructions by the various tools for an additional 3 simulated CRISPRs( Supplementary file 3 to 6).

Line 275: “data not shown” is replaced by “Table 4” concerning the spacer 38 variant
Line 286: replace Go with Gb 🡪 We checked that the RAM was indeed 32 Go.
Line 362: Could it be easily adapted to other species? Re-write...not appropriate
🡪 we made explicit that the exhaustive explanation on this topic lies in the Methods section by adding « and described in detail in Section 4d». We let the following sentences explaining which tools can help for the set-up of the catalogue, especially CRISPRcas-finder.

Figures:

Largely fine, but would recommend modifying/edit text and font styles to look clearer and optimum.
🡪 Some color codes and police sizes were modified to improve clarity in Figure 1 and 3.

Typos:
The manuscript is generally well written, but a thorough read-through is needed as there are a number of typos, formatting and grammatical errors. Some of which I have highlighted above.

Discussion:
I would like to see a little more about how this software has resolved the frequency and nature of IS6110 insertions in the CRISPR locus. Also, how does the software cope with large scale deletions of the locus due to IS6110?

🡪We fully agree that this topic is very interesting to better understand TB genome evolutionary dynamics. As this article focuses on the complex issue of explaining the reliability and usefullness of CRISPRbuilder-TB, we added only a few sentences in the paragraph compairing CRISPRbuilder-TB with other tools, pointed towards our companion article further describing TB-CRISPR evolutionary dynamics :

« *In contrast, CRISPR-builder TB could retrieve IS*6110 *sequences insertion positions as shown on strains for which reliable complete genome sequences were available (for instance H37Ra, L4.9,* ***Table 2, Supplemental File 10****). On other SRR, CRISPRbulder-TB retrieved between 0 and 2 copies of IS6110 in the CRISPR locus, in either orientation (****Supplementary file 2****, sheet “IS”, and see also companion article Refrégier et al [1] or its reviewed version soon to be published).* ».
