# Supplementary material for: CRISPRbuilder-TB: “CRISPR-builder for tuberculosis”. Exhaustive reconstruction of the CRISPR locus in mycobacterium tuberculosis complex using SRA
Source: PLoS Comput Biol. 2021 Mar 5;17(3):e1008500. doi: 10.1371/journal.pcbi.1008500 (PMC7968741; doi:10.1371/journal.pcbi.1008500)
Supplement: Attachment — Submitted filename: Answer to reviewers Plos Comp_nov2020.docx [file pcbi.1008500.s014.docx]

**Reviewer's Responses to Questions. (Nov. 2^nd^, 2020, R2)**

**Comments to the Authors:
Please note here if the review is uploaded as an attachment.**

*Answer : Yes, the review is uploaded as an attachment.*

Reviewer #1: The authors have successfully addressed the following major comments:

- Detailed documentation on how to download, install and run their tool on GitHub has been now provided.
- They argued well why the used short reads as the tool’s input, and not assemblies, and thus the choice of CRISPR predictor tools they used to compare their tool’s predictions against.

- Added some discussion on the functionality of CRISPR locus in MTC.

*We thank the reviewer and are glad to see that he acknowledged improvements on these major issues of our revision.*

However, the authors haven’t addressed the following point:

The newly added Supplementary_File3.odt, Supplementary_File4.odt and Supplementary_File5.odt do not provided

“A simple visual representation of simulated and reference vs. reconstructed CRISPR loci is needed to easily identify and compare regions of homology, order of CRISPR features (DR, spacers) and variants (SNPs, duplications, IS6110) between simulated and reference compared to reconstructed loci.”

*Answer to the reviewer : we thank the reviewer and we created a visual display of simulated CRISPR using CRASS, CRISPR detector and CRISPRbuilder TB and provided in Supplementary File 4.*

*Former Supplementary Files 3,4,5 are now merged in a single Supplementary File 3*

All minor comments were successfully addressed.

Reviewer #2: None
